# Improving the computational efficiency of fully Bayes inference and assessing the effect of misspecification of hyperparameters in whole-genome prediction models

**DOI:** 10.1186/s12711-015-0092-x

**Published:** 2015-03-07

**Authors:** Wenzhao Yang, Chunyu Chen, Robert J Tempelman

**Affiliations:** Department of Animal Science, Michigan State University, East Lansing, MI 48824-1225 USA

## Abstract

**Background:**

The reliability of whole-genome prediction models (WGP) based on using high-density single nucleotide polymorphism (SNP) panels critically depends on proper specification of key hyperparameters. A currently popular WGP model labeled BayesB specifies a hyperparameter *π*, that is `loosely used to describe the proportion of SNPs that are in linkage disequilibrium (LD) with causal variants. The remaining markers are specified to be random draws from a Student *t* distribution with key hyperparameters being degrees of freedom *v* and scale *s*^2^.

**Methods:**

We consider three alternative Markov chain Monte Carlo (MCMC) approaches based on the use of Metropolis-Hastings (MH) to estimate these key hyperparameters. The first approach, termed DFMH, is based on a previously published strategy for which *s*^2^ is drawn by a Gibbs step and *v* is drawn by a MH step. The second strategy, termed UNIMH, substitutes MH for Gibbs when drawing *s*^2^ and further collapses or marginalizes the full conditional density of *v*. The third strategy, termed BIVMH, is based on jointly drawing the two hyperparameters in a bivariate MH step. We also tested the effect of misspecification of *s*^2^ for its effect on accuracy of genomic estimated breeding values (GEBV), yet allowing for inference on the other hyperparameters.

**Results:**

The UNIMH and BIVMH strategies had significantly greater (*P* < 0.05) computational efficiencies for estimating *v* and *s*^2^ than DFMH in BayesA (*π* = 1) and BayesB implementations. We drew similar conclusions based on an analysis of the public domain heterogeneous stock mice data. We also determined significant drops (*P* < 0.01) in accuracies of GEBV under BayesA by overspecifying *s*^2^, whereas BayesB was more robust to such misspecifications. However, understating *s*^2^ was compensated by counterbalancing inferences on *v* in BayesA and BayesB, and on *π* in BayesB.

**Conclusions:**

Sampling strategies based solely on MH updates of *v* and *s*^2^, and collapsed representations of full conditional densities can improve the computational efficiency of MCMC relative to the use of Gibbs updates. We believe that proper inferences on *s*^2^, *v* and *π* are vital to ensure that the accuracy of GEBV is maximized when using parametric WGP models.

**Electronic supplementary material:**

The online version of this article (doi:10.1186/s12711-015-0092-x) contains supplementary material, which is available to authorized users.

## Background

Genomic predictions based on the use of high-density single nucleotide polymorphisms (SNPs) distributed across the genome have been increasingly adopted for animal and plant breeding programs. Parametric Bayesian methods have become particularly popular, most notably BayesA and BayesB, which were first introduced by Meuwissen *et al*. [1]. BayesB specifies a mixture prior on the SNP specific effects, with a point mass at 0 with probability (1- *π*), or randomly drawn, with probability *π*, from a Student *t* distribution with degrees of freedom *v* and scale parameter *s*^*2*^; BayesA is BayesB with *π* = 1. Note that Meuwissen *et al*. [1] define *π* in the opposite manner, whereas we prefer the definition of *π* that is used most commonly in the variable selection literature [2]. Hence, *π* is typically believed to be the proportion of SNPs that are associated or in linkage disequilibrium (LD) with causal variants, although this interpretation is somewhat complicated by varying degrees of LD across the genome. These hyperparameters (*v*, *s*^*2*^ and *π*) are relevant in that they partly characterize the genetic architecture of traits, but more importantly depend upon the density or characteristics of the SNPs used in the analyses [3].

Key hyperparameters in BayesA or BayesB whole-genome prediction (WGP) models have been arbitrarily specified in a wide selection of WGP studies. Meuwissen *et al*. [1] used arguments based on population genetics to set *v* and *s*^*2*^ at 4.2 and 0.04, respectively, in the BayesB model of their simulation study. In various simulation and/or empirical data analysis scenarios, it seems necessary to carefully tune key hyperparameters, since their optimal specifications should depend upon several key factors. For example, one could loosely interpret *s*^*2*^ as a “typical” SNP effect variance component in a BayesA model (based on the *t*-distribution being defined as a scaled-inverted chi square distribution on SNP-specific variances with heterogeneity across SNPs determined by *v*), such that its value should be inversely related to the number of SNPs used in an analysis. We have demonstrated this previously [3].

Inference in BayesA- or BayesB-like models is conducted using either Markov chain Monte Carlo (MCMC) methods for fully Bayesian inference or using faster, albeit approximate, methods based on the use of the expectation maximization (EM) algorithm or its various derivatives [4]. Unfortunately, it has not been readily established how to properly infer these hyperparameters in the EM-based methods, such that they are often arbitrarily “tuned” or specified [5]. Furthermore, although it is possible to infer these same hyperparameters using MCMC, the poor efficiency and speed of these implementations have seemingly discouraged their use [6], although recently some software has facilitated formal Bayesian inference on at least some of these hyperparameters [7]. In particular, it has been noted that the correlation between *v* and *s*^*2*^ across MCMC cycles is generally so large that these two hyperparameters are nearly non-identifiable from each other [6,8]. A MCMC strategy for a “fully” BayesA model was presented by Yi and Xu [9] who applied a Gibbs update for the full conditional density (FCD) on *s*^*2*^, since the conditional conjugate is a Gamma prior, whereas a Metropolis Hastings (MH) update was used on sampling from the FCD of *v*, since it is not recognizable [9]. We label this particular algorithm as DFMH (i.e., sampling *v* using MH), and it is the control or reference strategy in this paper.

Computational efficiency in MCMC schemes is related to the degree of mixing or autocorrelation between subsequent samples of the same parameter. The most popular metric for determining the degree of mixing or autocorrelation for a fixed number of MCMC cycles on a particular parameter is the effective sample size (ESS), which can be readily computed using software packages like CODA [10]. The ESS determines the effective number of independent draws such that a greater degree of autocorrelation between subsequent samples for the same parameter would lead to a smaller ESS and hence poorer computational efficiency. Now, although there are clear exceptions, MCMC sampling strategies that lead to a greater ESS for a certain total number of MCMC cycles tend to have greater computational cost per cycle. This realization is reflected in other quantitative genetics applications [11,12] that derived various metrics to integrate these two components of computational efficiency.

We surmised that, compared to the use of DFMH, there may be a number of MCMC strategies that could improve the computational efficiency of inferring upon key hyperparameters in a BayesA or BayesB WGP model. Furthermore, the efficiency of any strategy could markedly depend on the choice of an appropriate scale for the parameters being sampled. For example, a highly nonlinear relationship between two variables can often be rendered linear by transforming either one or both of the corresponding parameters. When *v* and *s*^*2*^ are both log-transformed, the resulting scatterplot of MCMC samples of the transformed variables against each other often demonstrates a more linear relationship, although we have also observed counterexamples, where a linear relationship is maintained on the original scale. At any rate, a change of variable might facilitate potentially more efficient MCMC sampling strategies based on multivariate Gaussian proposal densities, for example, in Metropolis sampling schemes [13].

There were two primary objectives in this study. First, we wanted to explore alternative strategies to improve the computational efficiency of estimating hyperparameters in BayesA and BayesB WGP models. Second, given the prevalent practice of specifying rather than estimating these hyperparameters, we wanted to assess the impact of misspecifying *s*^*2*^ on the accuracy of genomic estimated breeding values (GEBV) or even whether *s*^*2*^ could be reasonably extrapolated based on analyses derived from other marker densities.

## Methods

### Whole-genome prediction model

The WGP model used for comparison of the various computational strategies and/or hyperparameter specifications can be denoted as follows:

1 $$ {y}_i={\mathbf{x}}_i^{\hbox{'}}\boldsymbol{\upbeta} +{\displaystyle \sum_{j=1}^m{z}_{ij}}{g}_j+{e}_i, $$

where *y*_*i*_ is the phenotype for animal *i* (*i* = 1,2,…,*n*), **β** is a vector of fixed effects such that ${\mathbf{x}}_i^{\hbox{'}}$ is the known incidence row vector connecting *y*_*i*_ to **β**, *z*_*ij*_ is the genotype covariate for SNP *j* on animal *i*, coded as either 0, 1, or 2 copies of a reference allele for SNP *j* on animal *i*, *g*_*j*_ is the random effect for SNP *j*, and *e*_*i*_ is the residual. We assume ${g}_j\sim NID\left(0,{\sigma}_{g_j}^2\right)$ conditionally and ${e}_i\sim N\left(0,{\sigma}_e^2\right)$.

Stranden and Christensen [14] recently demonstrated that MCMC mixing of key parameters, including fixed effects, can be dramatically improved by simply expressing the covariate *z*_*ij*_ for each SNP genotype *j* as a deviation from that SNP average; i.e. using ${z}_{ij}*={z}_{ij}-\frac{1}{n}{\displaystyle \sum_{i=1}^n{z}_{ij}}$ as the covariate in Model (1). As Stranden and Christensen [14] further demonstrate, this recoding does not alter inference on SNP effects {*g*_*j*_}; however, we assumed that this recoding could only help to facilitate mixing on all other parameters whose FCD are functions of {*g*_*j*_}. Hence, we applied this strategy for all computational efficiency comparisons considered in this paper.

The prior distribution of ${\sigma}_{g_j}^2$ in BayesB is a mixture of two components: a scaled inverted chi-square distribution with ${\sigma}_{g_j}^2\sim {\chi}^{-2}\left(\nu, \nu {s}^2\right)$, having probability *π*, and a spike at 0 with probability (1 − *π*). Here, *π* loosely represents the proportion of SNPs having associated genetic effects on the phenotype. Recall that BayesA is a special case of BayesB when *π* = 1. Following Yang and Tempelman [3], we specify the following prior distributions on the hyperparameters: *ν* ~ *p*(*ν*) ∝ (*ν* + 1)^− 2^ in both BayesA and BayesB, the Gelman prior [15] *s*^2^ ~ *χ*^− 2^(−1, 0) in BayesA, a proper but diffuse conjugate prior *s*^2^ ~ *Gamma*(*α*_*s*_, *β*_*s*_) such that $\operatorname{E}\left({s}^2\right)\sim \frac{\alpha_s}{\beta_s}$ with shape parameter *α*_*s*_ = 0.1 and rate parameter *β*_*s*_ = 0.1 in BayesB; more informative alternative specifications could be provided for *s*^2^ in either model as desired. Finally, we specify *π* ~ *p*(*π*|*α*_*π*_, *β*_*π*_) = *Beta*(*α*_*π*_, *β*_*π*_) with *α*_*π*_ = 1 and *β*_*π*_ = 8 in BayesB. A Beta prior is equivalent to specifying a prior mean of *α*_*π*_/(*α*_*π*_ + *β*_*π*_) based on a prior sample size of *α*_*π*_ + *β*_*π*_. For all three computational strategies that we describe subsequently, we adapt the same commonly used MCMC strategies for sampling from all parameters or random variables as outlined by Meuwissen *et al*. [1], noting the exceptions for *v*, *s*^2^, and *π* that Meuwissen *et al*. [1] treated as fixed or known. For all algorithms, the FCD of *π* in Bayes B was deemed to be *Beta*(*α*_*π*_ + *m*_1_, *β*_*π*_ + *m* − *m*_1_), where *m*_*1*_ denotes the number of non-zero *g*_*j*_ sampled for that MCMC cycle [3]. We now describe each of three computational strategies.

### Univariate metropolis Hastings sampling on *v* and Gibbs update on *s*^2^ (DFMH)

This strategy, which we designate as DFMH, closely follows Yi and Xu [9]. The FCD of *v* does not have a recognizable form; hence sampling from this FCD requires a strategy other than a Gibbs step. Here, we used the MH algorithm to sample from the FCD of *v*, drawing from our experiences in various other applications [3,16-18]. More specifically, we generate from the FCD of *ξ* = log(*ν*), ensuring that the FCD of *ξ* takes into account the Jacobian of the transformation from *v* to *ξ* [See Additional file 1]. Since *ξ* can conceptually be defined anywhere on the continuous real line, we believe that this transformation better justifies the use of a Gaussian proposal density centered on the value of *ξ* from the previous MCMC cycle; i.e., a random walk MH step [19]; alternatively, a heavier-tailed Student *t* proposal density could be used as well. During the first half of burn-in, we adaptively tune the variance of this proposal density such that the MH acceptance ratios are intermediate (i.e., 25 to 75%), adapting the strategy described by Muller [20] and in accordance with standard recommendations [21,22]. The variance of the proposal density was then fixed for the last half of burn-in, in order to ensure a proper convergent MCMC algorithm. Yi and Xu [9] demonstrated that the FCD of *s*^2^ is Gamma, provided that a conditionally conjugate Gamma prior is used. Hence, for DFMH, we sampled *v*, using the described MH update, and *s*^2^ with a Gibbs update. To facilitate even better the mixing of *v* through the joint posterior density, given that the MH acceptance rates are potentially less than 50%, we drew 10 MH samples for *v* per MCMC cycle conditional on all other unknowns. Further details on the DFMH strategy are provided in [See Additional file 1].

### Univariate metropolis hastings sampling for each of *v* and *s*^2^ (UNIMH)

Metropolis Hastings sampling, if properly tuned with good proposal densities and intermediate acceptance rates, can often lead to faster mixing and hence greater MCMC efficiency relative to Gibbs sampling. This is because MH sampling typically proposes bigger jumps throughout the posterior density compared to the use of Gibbs sampling. Hence, we propose a second strategy, UNIMH, whereby we again use MH to sample from *v* but also use MH to sample from *s*^2^. However, we also embed an additional strategy, whereby the FCD for both *v* and *s*^2^ is based on integrating out ${\left\{{\sigma}_{g_j}^2\right\}}_{j=1}^m$ from the joint posterior density of all unknowns, such that the prior specification on g is explicitly provided as a mixture involving a Student *t* distribution, rather than a scaled inverted chi-square distribution mixture of normals. This strategy leads to a “collapsed” sampler, which is also known to facilitate faster MCMC mixing [23] since uncertainty on ${\left\{{\sigma}_{g_j}^2\right\}}_{j=1}^m$ is completely accounted for in the MCMC draws of *v* and *s*^2^.

As with *v* in DFMH, we sample *s*^2^ by invoking a change of variable to its logarithm (i.e., *ψ* = log(*s*^2^)), allowing for the Jacobian of the transformation from *s*^2^ to *ψ*, and use a random walk MH algorithm based on a Gaussian proposal density for *ψ*. Similar to what was done for *v*, the variance of this proposal density was only tuned for intermediate acceptance rates during the first half of burn-in, to ensure a properly convergent MCMC chain. For reasons similar to those provided for DFMH, 10 MH samples per MCMC cycle were specified for sampling *v* and *s*^2^ in UNIMH. Further details on this strategy are provided in [See Additional file 1].

### Bivariate metropolis hastings sampling on *v* and *s*^2^ (BIVMH)

As previously noted, the posterior correlation between *v* and *s*^2^ can be high; hence, it might be advantageous to jointly sample these two parameters with a bivariate random walk MH sampler, as demonstrated with a different application by Ntzoufras [13]. Hence, we proposed a third sampling algorithm that we named BIVMH. Here, we divided the burn-in period for this strategy into four stages of equal lengths with respect to the number of MCMC cycles; arguably, a more efficient partition of these stages might be possible given that these stages may not necessarily need to be of the same length. In Stage 1, we sampled log(*v*) and log(*s*^2^) from their respective FCD using the UNIMH strategy previously described, fine-tuning the variances of the two separate Gaussian proposal densities to ensure that MH acceptance rates fall between 25 and 75%. In Stage 2, we sampled log (*v*) and log (*s*^2^) using UNIMH, fixing the variances of their respective proposal densities to those values tuned at the end of Stage 1, while computing the empirical correlation between the samples of log (*v*) and log (*s*^2^) drawn from within the same cycle. In Stage 3, log (*v*) and log (*s*^2^) were jointly sampled using a bivariate Gaussian proposal density with variances based on those tuned at the end of Stage 1 and a covariance based on the correlation computed from Stage 2. During Stage 3, we further fine-tuned the proposal variances to ensure intermediate acceptance rates for joint samples of log (*v*) and log (*s*^2^), with the proposal covariance based on the same correlation derived in Stage 3. In Stage 4, we drew samples using the same joint MH random walk from the newly tuned bivariate Gaussian proposal density in Stage 3 but without further tuning, in order to ensure a properly convergent MCMC chain before the end of the burn-in period. At the end of Stage 4, and hence burn-in, we saved samples for the hyperparameters of *v* and *s*^2^ (i.e., back-transformed) for MCMC-based fully Bayesian inference. As with UNIMH, 10 MH samples were drawn for each of *v* and *s*^2^ at each stage per MCMC cycle. Details on this strategy are provided in [See Additional file 1].

### Simulation study

In order to test the computational efficiency of the three sampling strategies, DFMH, UNIMH and BIVMH, under the BayesA and BayesB modeling specifications, we simulated 15 replicated datasets using the HaploSim package in R [24]. The simulated genome was composed of one chromosome of 1 Morgan that comprised 100 000 equally spaced loci. For each of the 100 animals in the base population, every 5^th^ locus on this chromosome was heterozygous (i.e., for a total of 20 000 such loci), whereas the remaining 80 000 loci were completely monomorphic, similar to the simulation in Coster *et al*. [24]. Individuals were randomly mated to generate 100 animals within each of 6000 subsequent generations in order to generate LD between loci. The number of recombinations per meiosis was drawn from a Poisson distribution, with the position of each recombination being randomly drawn from a uniform distribution on the chromosome consistent with a Haldane mapping function (i.e., no interference). Furthermore, we specified the recurrent mutation rate to be 10^−5^ per locus per generation.

In Generation 6000, random matings were used to increase the population size to 1000 individuals in Generation 6001. In Generation 6001, we deleted loci with a minor allele frequency (MAF) less than 0.05 and randomly selected 30 from the remaining loci to be quantitative trait loci (QTL). Following Meuwissen *et al*. [1], we simulated allele substitution effects α for these 30 QTL from a reflected Gamma distribution with shape parameter 0.4 and rate parameter 1.66, such that the true breeding values (TBV) were genotype-based linear combinations of α. Phenotypes for animals in Generation 6001 were generated based on a heritability of 50%, i.e., such that ${\sigma}_e^2$ = var(TBV). Note that the heritability specification thereby renders the specification of the rate parameter value of 1.66 irrelevant. Additionally, genotypes for 1000 offspring in Generation 6002 were based on random matings of individuals in Generation 6001. Again, TBV were based on linear combinations of α based on QTL genotypes inherited from Generation 6001.

After discarding SNPs with a MAF less than 0.05, we then selected every single, 4^th^ and 10^th^ SNP for inclusion in analyses in order to consider the effect of different marker densities; i.e., high (average of 2394 SNPs), medium (average of 598 SNPs), and low (average of 239 SNPs) across the 15 replicates; these marker densities subsequently influence the average pairwise LD measures between adjacent markers. We compared the computational efficiencies between the three strategies for inferring on key hyperparameters (i.e., *π* , *v* and *s*^*2*^) under these three marker densities. We ignored additionally fitting polygenic effects [25] for all comparisons in the simulation study to further facilitate computational feasibility, assuming that this would not affect the relative efficiency of each computing strategy.

We compared the computational efficiencies of the three MCMC strategies for each replicated dataset, considering each marker density. Since computational efficiency pertains to a particular hyperparameter, it was defined as the effective sample size (ESS) for the post-burn-in MCMC cycles divided by total CPU time, i.e., ESS/CPU recorded in number per second. That is, the greater ESS/CPU, the greater the computational efficiency for inferring the posterior density of that particular hyperparameter.

Recognizing that many researchers do not infer some or even all hyperparameters in WGP models, because of the perceived inferential challenges, we thought it was important to assess the impact of misspecification of these parameters on the accuracy of genomic prediction. Using the same simulated data as described previously, we focused on five scenarios, all at the medium marker density (selecting every 4^th^ marker). Each scenario was based on setting *s*^*2*^ as an arbitrary multiplicative constant of the average posterior mean at the medium marker density (${\overline{s}}_{med}^2$), based on the corresponding model (BayesA or BayesB) while other hyperparameters (*v* and *π* where applicable) were inferred upon. Values for *s*^*2*^ for these five scenarios were set to 1) *s*^*2*^ = ${\overline{s}}_{med}^2$, 2) *s*^*2*^ = 0.1 ${\overline{s}}_{med}^2$, 3) *s*^*2*^ = 0.01 ${\overline{s}}_{med}^2$, 4) *s*^*2*^ = 10 ${\overline{s}}_{med}^2$, and 5) *s*^*2*^ = 100 ${\overline{s}}_{med}^2$. Note that the specification of ${\overline{s}}_{med}^2$ depended on which model (BayesA or BayesB) was used, as described later, and that the other hyperparameters (*v* and *π* where applicable) were still inferred upon within each of these scenarios in order to remove confounding effects of their misspecification.

We also investigated if it was possible to specify a roughly good working value for *s*^*2*^ by merely extrapolating it from an estimate derived from, e.g., the analysis of the same phenotypes but based on a SNP panel with a different marker density. Since *s*^*2*^ represents a typical value for the SNP-specific variances ${\sigma}_{g_j}^2$, with the mean being greater and the mode being less than *s*^*2*^, the value of *s*^*2*^ should be inversely related to the number of SNPs, as previously reported [3]. For example, given that there were, on average, four times as many markers in the high-density panel as there were in the medium-density panel in our simulation study, a candidate specification for *s*^*2*^ at the high-density might be to use *s*^2^ = 0.25 ${\overline{s}}_{med}^2$. Similarly, since there were 2.5 times as many SNPs in the medium-density panel than in the low-density panel, a candidate specification for the low-density marker specification might be *s*^2^ = 2.5 ${\overline{s}}_{med}^2$. These specifications for *s*^2^ were compared for their effect on the accuracy of GEBV relative to the situation where *s*^2^ was directly inferred upon under BayesA and BayesB for all 15 replicated datasets. Again, all these comparisons were conducted such that all remaining hyperparameters (*v* and *π* where applicable) were inferred upon as well.

In all cases, accuracy of prediction was defined as the correlation between GEBV and TBV, where GEBV for animal *i* was defined as ${\displaystyle \sum_{j=1}^m{z}_{ij}^{*}}{\overline{g}}_j$ , where ${\overline{g}}_j$ is the posterior mean of *g*_*j*_ and TBV is defined as before. Hypothesis testing between accuracies of different methods were based on blocking on replicated datasets, using a Wilcoxon signed rank test. Furthermore, every single MCMC analysis was based on 120 000 cycles during burn-in, followed by 400 000 cycles after burn-in, saving every 10 for a total of 40 000 samples.

### Data application

In the real dataset, 2296 mice were genotyped for 12 147 SNPs with a high pairwise LD of *r*^*2*^ = 0.6 [26]. After filtering data on genotypes [3], there were 1940 animals with 10 467 SNPs. We selected 50, 100 and 200 SNPs from each of the 19 autosomes to create three different marker densities using pre-corrected body weight at 6 weeks as phenotypes. As in Yang and Tempelman [3], we also modeled the random effects of cage in addition to SNP effects and polygenic effects in the WGP model, using the Gelman prior [15] for the cage (${\sigma}_c^2$) and the polygenic (${\sigma}_u^2$) variance components. After merging phenotypes with the genotypes, 1917 animals remained with complete phenotypes and genotypes on 950, 1900 and 3800 SNPs for the three different marker densities across the 19 autosomes. Computing efficiency was directly compared between each of the three methods based on 80 000 samples obtained by saving every 10^th^ sample of 800 000 MCMC cycles, after a burn-in period of 120 000 samples.

## Results

### Simulation study

By selecting every single, 4^th^ and 10^th^ SNP for inclusion, the average LD measure (*r*^2^) between adjacent SNPs, was 0.32, 0.24 and 0.17 for the three marker densities over the 15 replicated datasets. Pairwise scatterplots used to assess quality control on our procedures are in [See Additional file 2: Figures S1, S2, S3, S4 and S5]. Inferences on *s*^2^ using each of the three sampling strategies DFMH, UNIMH and BIVMH were compared under both BayesA [See Additional file 2: Figure S1] and BayesB [See Additional file 2: Figure S2] specifications. We observed that estimates (posterior medians) of *s*^2^ decreased as the marker density increased and that estimates derived from BayesB were generally one order of magnitude greater than those in BayesA. Furthermore, posterior medians of *π* generally increased [See Additional file 2: Figure S3], whereas posterior medians of *v* generally decreased as marker density increased, for both BayesA [See Additional file 2: Figure S4] and BayesB [See Additional file 2: Figure S5].

Overall, pairwise scatterplots of the posterior medians of *s*^2^, *π* and *v* under the three computational strategies for each of the three marker densities indicated very good agreement, as expected, since the joint posterior density should not differ regardless of whether one uses DFMH, UNIMH or BIVMH, provided that the prior specifications are identical. Better agreement between the three algorithms was generally found for BayesA than for BayesB inference on *s*^2^ and *v*. This was as anticipated, since BayesB requires inference on one more hyperparameter (π) and hence potentially greater Monte Carlo error for the same number of cycles; furthermore, inference on *s*^2^ and *v* is essentially based on information from only non-zero SNP effects, which is appreciably less than the effective total number of SNPs used for estimating *s*^2^ and *v* in BayesA [3]. Moreover, greater agreement was found between all algorithms at the lowest-density of markers (last row of each scatterplots in Figures S1, S2, S3, S4 and S5 [See Additional file 2: Figures S1, S2, S3, S4 and S5], since the ESS were generally much greater (results not shown) in that case; that is, higher ESS generally translates into smaller Monte Carlo error. For the same reason, there was generally greater agreement between UNIMH and BIVMH (last column of each scatterplots in Figures S1, S2, S3, S4 and S5 [See Additional file 2: Figures S1, S2, S3, S4 and S5], since the DFMH showed relatively poorer MCMC mixing (low ESS) and hence greater Monte Carlo error.

Table 1 provides median ESS/CPU as a measure of MCMC computational efficiency for each of the three strategies under each of the three marker densities for *v* and *s*^2^, respectively, under the BayesA model. In all cases, ESS/CPU increased with lower marker densities, since that leads to an inferential situation of more data information per marker (i.e., higher *n*/*m*) or a greater level of determinedness [27]. Furthermore, ESS/CPU was always higher for BIVMH and UNIMH compared to DFMH (*P* < 0.05). For *v* at the high marker density (r^2^ = 0.32), UNIMH had higher ESS/CPU than BIVMH (*P* < 0.05), whereas for *s*^2^ at the medium marker density (r^2^ = 0.24), median ESS/CPU was higher for BIVMH than UNIMH (*P* < 0.05). Interestingly, the ratios of computing efficiencies for both hyperparameters between UNIMH and BIVMH with DFMH hovered between 3 and 6 across marker densities. Efficiencies for the three alternative sampling strategies were also compared under the BayesB model for *v*, *s*^2^, and *π* (Table 2). Again, we found that UNIMH and BIVMH had significantly greater computing efficiencies compared to DFMH for all three hyperparameters (*P* < 0.05), with values several times greater for *v* and *s*^2^_,_ whereas the computing efficiency advantage was somewhat less pronounced for *π*.

**Table 1:** **Median computational efficiencies for inferring hyperparameters in BayesA based on alternative algorithms**

		**Computing efficiency** ^**2**^ **by algorithm**		
**Parameter**	**Density** ^**1**^	**DFMH**	**UNIMH**	**BIVMH**
*s* ^*2*^	0.17	0.065^a 2,3^	0.32^b^	0.37^b^
	0.24	0.014^a^	0.058^b^	0.063^c^
	0.32	0.0015^a^	0.0056^b^	0.0043^b^
ν	0.17	0.08^a^	0.42^b^	0.47^b^
	0.24	0.016^a^	0.064^b^	0.069^b^
	0.32	0.00088^a^	0.0035^b^	0.0026^c^

^1^Based on average r^2^ between adjacent markers; ^2^computing efficiency measured as effective sample size divided by total CPU time in seconds; ^3^any two medians not sharing the same letter within the same row are concluded to be different from each other based on a Wilcoxon rank sum test (P<0.05).

**Table 2:** **Median computational efficiencies for inferring hyperparameters in BayesB based on alternative algorithms**

		**Computing efficiency** ^**2**^ **by algorithm**		
**Parameter**	**Density** ^**1**^	**DFMH**	**UNIMH**	**BIVMH**
*s* ^*2*^	0.17	0.080^a 2,3^	0.44^b^	0.50^c^
	0.24	0.033^a^	0.14^b^	0.15^b^
	0.32	0.0084^a^	0.032^b^	0.030^c^
ν	0.17	0.16^a^	1.10^b^	1.08^b^
	0.24	0.11^a^	0.85^b^	0.74^b^
	0.32	0.029^a^	0.42^b^	0.29^b^
*π*	0.17	0.12^a^	0.3^b^	0.29^b^
	0.24	0.029^a^	0.068^b^	0.071^b^
	0.32	0.0033^a^	0.0057^b^	0.0043^c^

^1^Based on average r^2^ between adjacent markers; ^2^computing efficiency measured as effective sample size divided by total CPU time in seconds; ^3^any two medians not sharing the same letter within the same row are concluded to be different from each other based on a Wilcoxon rank sum test (P<0.05).

**Figure 1:**
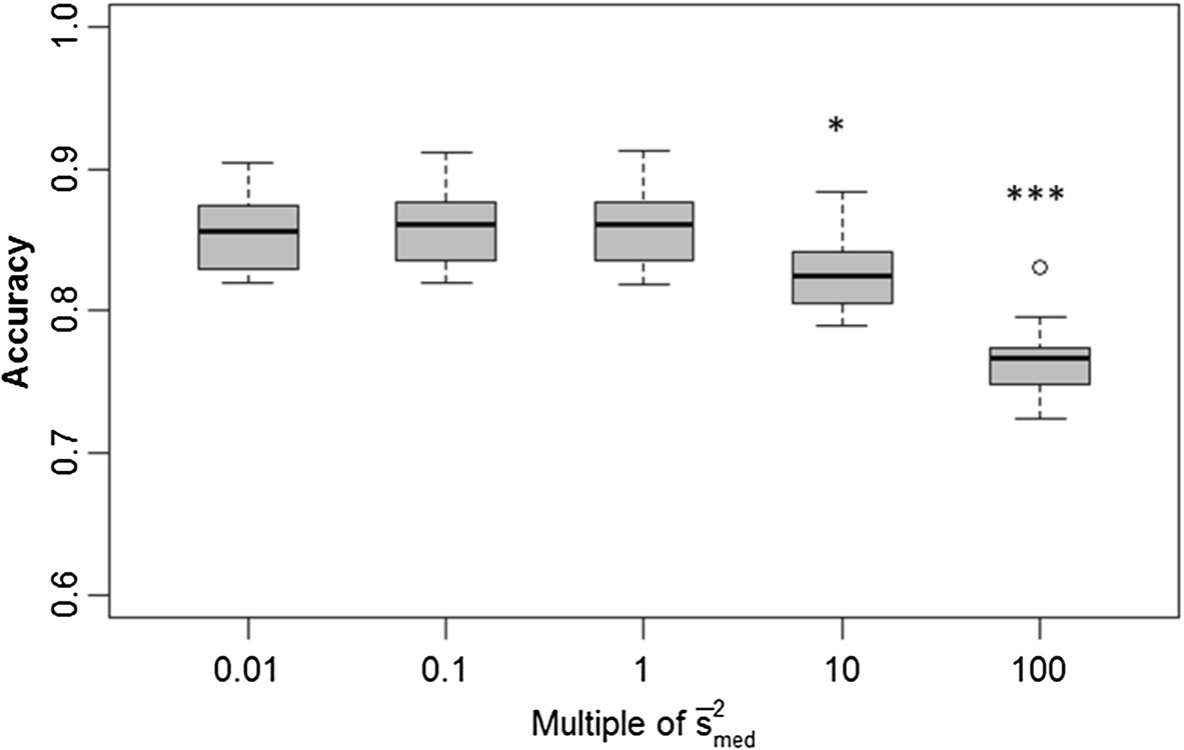
**Boxplots of breeding value accuracies under a BayesA model with** ***s*** ^**2**^ **set equal to different orders of magnitude.** *s* ^*2*^ is set to various multiples (0.01, 0.1, 1, 10 and 100) of its average posterior mean (${\overline{s}}_{med}^2$ = 7x10^−4^) as estimated from 15 replicates at an intermediate level of LD (adjacent pairwise r^2^ = 0.24); accuracies provided in the figure are based on those same 15 replicates; significant differences in accuracies from that based on 1 ${\overline{s}}_{med}^2$ are indicated by *(*P* < 0.01) or *** (*P* < 0.0001).

**Figure 2:**
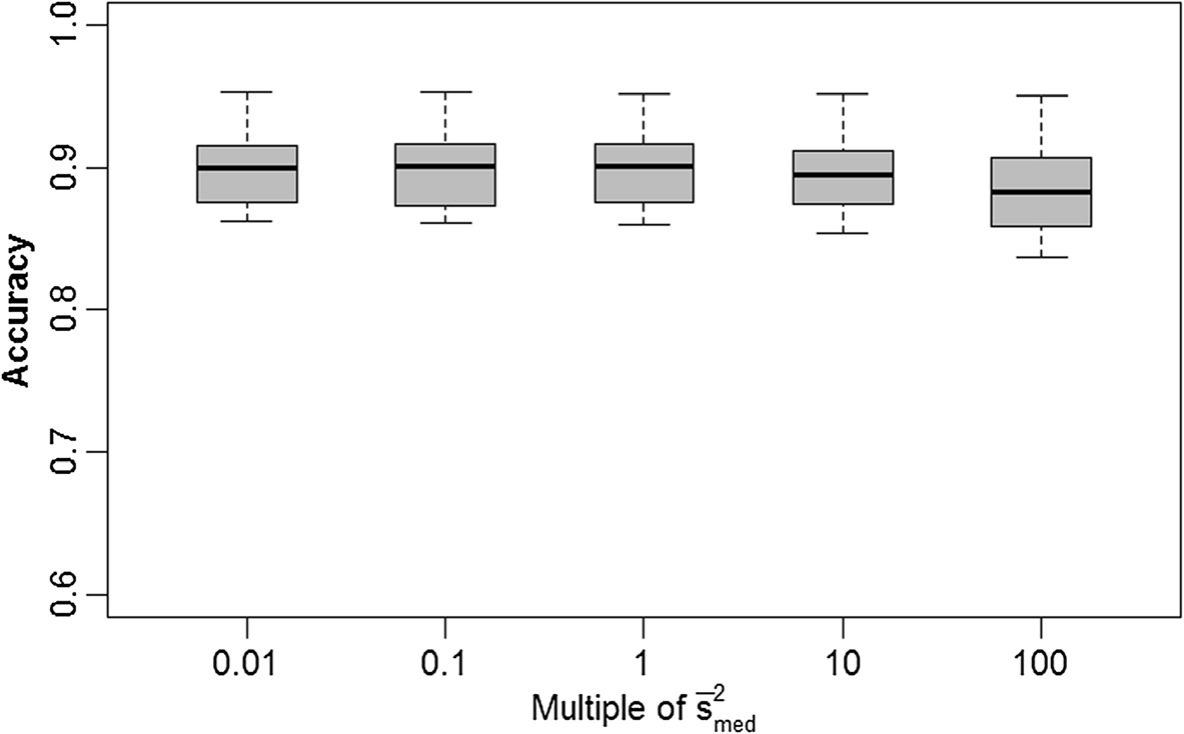
**Boxplots of breeding value accuracies under a BayesB model with** ***s*** ^**2**^ **set equal to different orders of magnitude.** *s* ^*2*^ is set to various multiples (0.01, 0.1, 1, 10 and 100) of its average posterior mean (${\overline{s}}_{med}^2$ = 4x10^−2^) as estimated from 15 replicates at an intermediate level of LD (adjacent pairwise r^2^ = 0.24); accuracies provided in the figure are based on the same 15 replicates.

We were interested in determining whether the accuracy of GEBV depends on misspecification of hyperparameters, say, *s*^2^. We assessed the impact on accuracy of GEBV based on setting *s*^2^ equal to a wide range of multiples (0.01× to 100×) of the average posterior mean (${\overline{s}}_{med}^2$ = 7×10^−4^ for BayesA, ${\overline{s}}_{med}^2$ = 4×10^−2^ for BayesB) across the 15 replicates under the medium marker density. For BayesA (Figure 1), we found no significant difference in accuracies when *s*^2^ was understated (i.e., *s*^2^ = 0.1 ${\overline{s}}_{med}^2$ and *s*^2^ = 0.01 ${\overline{s}}_{med}^2$); however, GEBV accuracies were significantly compromised when *s*^2^ was overstated, particularly at *s*^2^ = 100 ${\overline{s}}_{med}^2$ (*P* < 0.0001). For BayesB (Figure 2), we did not see any significant differences in accuracy of prediction between any of the various misspecifications of *s*^2^.

**Figure 3:**
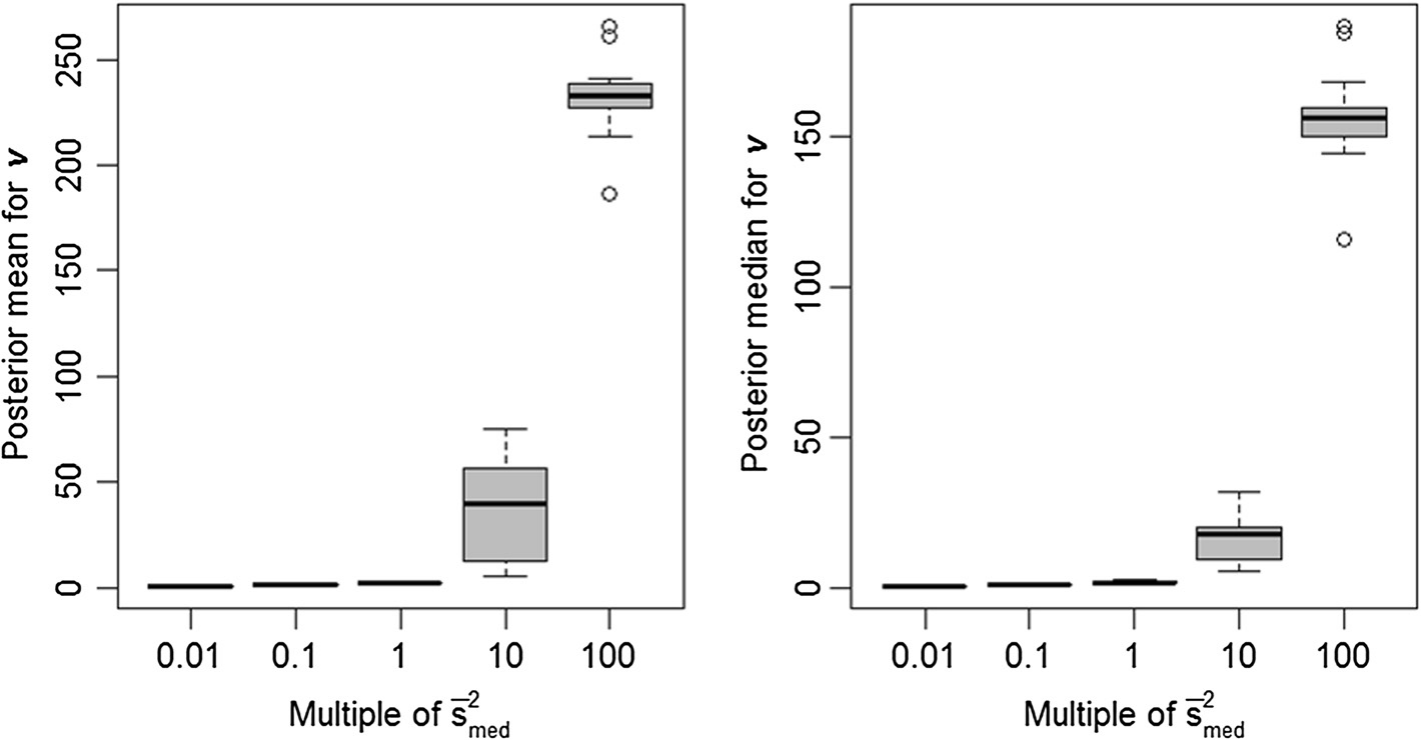
**Boxplots of posterior means and medians for** ***v*** **under the BayesA model with** ***s*** ^**2**^ **set equal to different orders of magnitude.** *s* ^*2*^ is set to various multiples (0.01, 0.1, 1, 10 and 100) of its average posterior mean (${\overline{s}}_{med}^2$ = 7x10^−4^) as estimated from 15 replicates at an intermediate level of LD (adjacent pairwise r^2^ = 0.24); estimates provided in the figure are based on the same 15 replicates.

**Figure 4:**
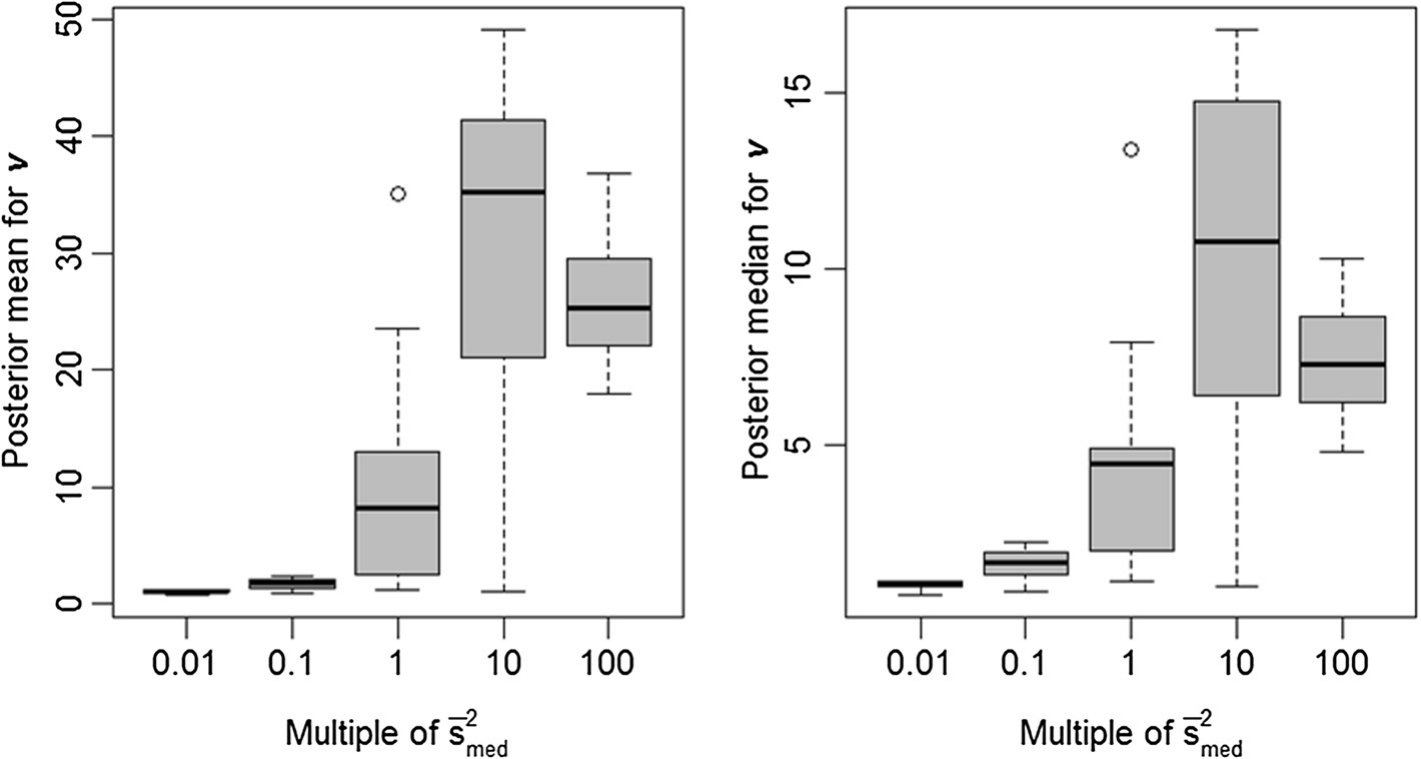
**Boxplots of posterior means and medians for** ν **under the BayesB model with** ***s*** ^**2**^ **set equal to different orders of magnitude.** *s* ^*2*^ is set to various multiples (0.01, 0.1, 1, 10 and 100) of its average posterior mean (${\overline{s}}_{med}^2$ = 4x10^−2^) as estimated from 15 replicates at an intermediate level of LD (adjacent pairwise r^2^ = 0.24); estimates provided in the figure are based on the same 15 replicates.

**Figure 5:**
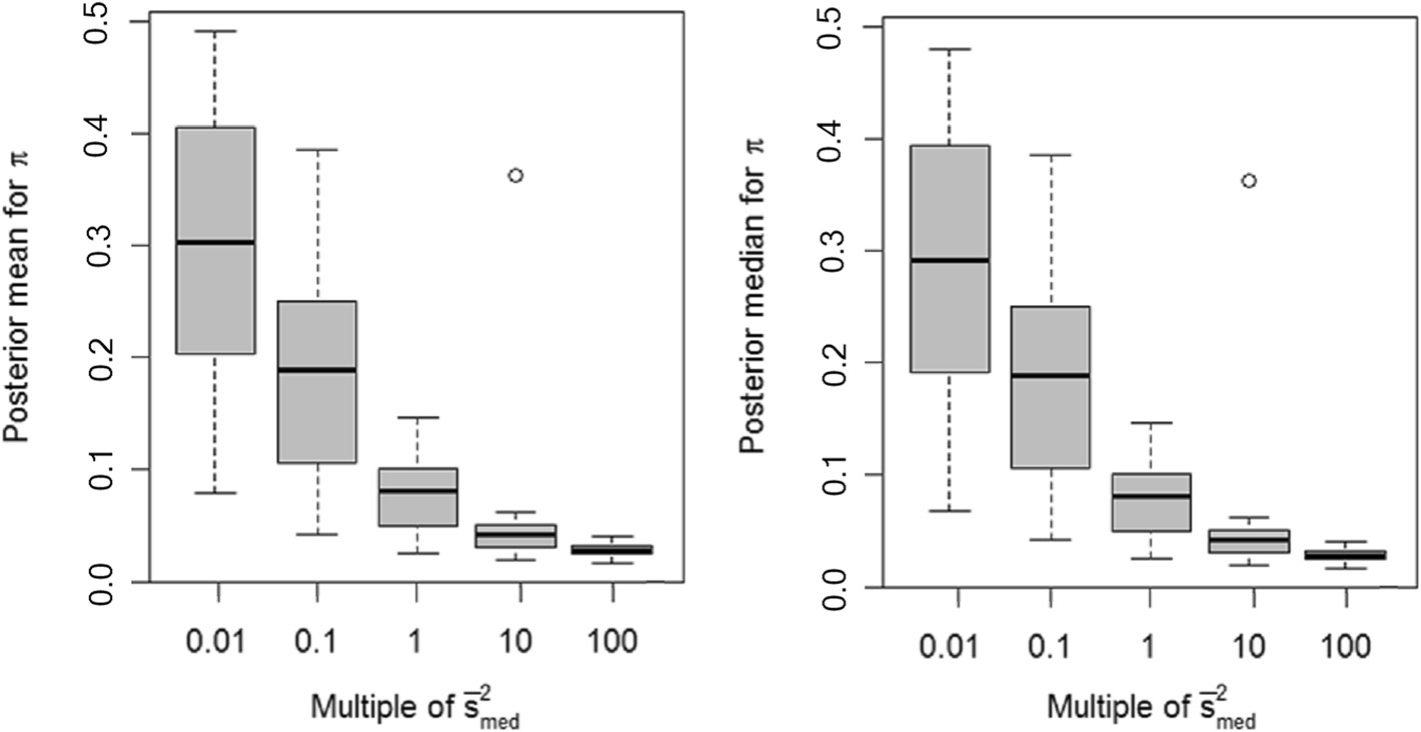
**Boxplots of posterior means and medians for** ***π*** **under the BayesB model with** ***s*** ^**2**^ **set equal to different orders of magnitude.** *s* ^*2*^ is set to various multiples (0.01, 0.1, 1, 10 and 100) of its average posterior mean (${\overline{s}}_{med}^2$ = 4x10^−2^) as estimated from 15 replicates at an intermediate level of LD (adjacent pairwise r^2^ = 0.24); estimates provided in the figure are based on the same 15 replicates.

Another question is whether some of the non-significant accuracy differences in these comparisons could be partly attributed to compensation in the inferences on other hyperparameters, specifically *v* and, additionally for BayesB, *π*. Indeed we noted that, as the specification on *s*^2^ increased from 0.01 ${\overline{s}}_{med}^2$ to 100 ${\overline{s}}_{med}^2$, the posterior means of *v* also increased under both BayesA (Figure 3) and BayesB (Figure 4), as somewhat anticipated given the high posterior correlation between these two hyperparameters. Misspecification of *s*^2^ also impacted estimates of *π* in a BayesB analysis, as illustrated in Figure 5. Nevertheless, this feature also provided BayesB with more flexibility than BayesA, as it pertains to misspecification of *s*^2^; that is, overstated values of *s*^2^ merely distribute the number of non-zero ${\left\{{g}_j\right\}}_{j=1}^m$ over a smaller number of markers, as indicated by lower estimates of *π*.

**Figure 6:**
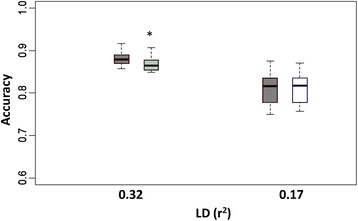
**Boxplots of breeding value accuracies under a BayesA model with** ***s*** ^**2**^ **either estimated or extrapolated from a different marker density.** Accuracies based upon 15 replicates at high and low levels of LD (pairwise LD r^2^ = 0.32 and 0.17). Dark grey boxplots pertain to BayesA inferring upon both *v* and *s* ^2^; extrapolations for *s* ^2^ based on average posterior mean (${\overline{s}}_{med}^2$ = 7x10^−4^) estimated from 15 replicates at a medium level of LD (pairwise LD r^2^ = 0.24) determined to be ${\overline{s}}_{med}^2$/4 for a high level of LD (light grey boxplot) and ${\overline{s}}_{med}^2$*2.5 for a low level of LD (white boxplot) while estimating ν; accuracies provided in the figure are based on the same 15 replicates at the respective LD levels and are judged different from each other based on *(*P* < 0.05).

**Figure 7:**
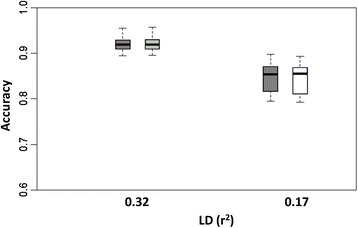
**Boxplots of breeding value accuracies under a BayesB model with** ***s*** ^**2**^ **either estimated or extrapolated from a different marker density.** Accuracies based upon 15 replicates at high and low levels of LD (pairwise LD r^2^ = 0.32 and 0.17). Dark grey boxplots pertain to BayesB inferring upon *π*, *v* and *s* ^2^; extrapolations for *s* ^2^ based on average posterior mean (${\overline{s}}_{med}^2$ = 4x10^−2^) estimated from 15 replicates at medium LD level (pairwise LD r^2^ = 0.24) determined to be ${\overline{s}}_{med}^2$/4 for high LD level (light grey boxplot) and ${\overline{s}}_{med}^2$*2.5 for low LD level (white boxplot) while estimating *π* and *v*, accuracies depicted in the figure are based on those same 15 replicates at the respective LD levels.

We also wondered if estimates of *s*^2^ based on analysis of certain marker densities could be extrapolated to other marker densities for analysis of the same phenotypes. Recall that ${\overline{s}}_{med}^2$ = 7×10^−4^ for BayesA, ${\overline{s}}_{med}^2$ = 4×10^−2^ for BayesB with the medium-density panel (r^2^ = 0.24). For the high-density panel that included four times as many markers, we specified *s*^2^ = 0.25 ${\overline{s}}_{med}^2$, whereas for the low-density panel, which contained only about 40% as many markers, we specified *s*^2^ = 2.5 ${\overline{s}}_{med}^2$. We found no significant differences in accuracies of prediction in any case (see Figures 6 and 7), except for a significantly lower accuracy for extrapolation on *s*^2^ at the higher marker density using BayesA (*P* = 0.04).

### Application to heterogeneous stock mice data

We summarize posterior inferences for the key hyperparameters under BayesA and BayesB analyses of the heterogeneous stock mice data in Tables 3, 4 and 5 for the three marker densities of 950, 1900 and 3800 SNPs, respectively. A fuller visual assessment of the posterior densities for each of these marker densities is provided in [See Additional file 2 Figure S6] for *s*^2^, in [See Additional file 2: Figure S7] for *v*, and for π in [See Additional file 2: Figure S8], which indicate general agreement between the three computing strategies, albeit there was seemingly a slight discrepancy for *v* between DFMH versus UNIMH and BIVMH under a BayesB model. Using the 950-SNP panel, the ESS/CPU for *s*^2^ was up to 3 times greater using UNIMH and BIVMH compared to DFMH, whereas the ESS/CPU for *v* was up to 50 times greater in UNIMH and BIVMH compared to DFMH. In other words, UNIMH and BIVMH had greater computing efficiency. For the 1900-SNP panel (Table 4), the ESS/CPU for UNIMH and BIVMH compared to DFMH were up to 80 times greater for *v* and up to 10 times greater for *s*^2^_,_ with advantages for *v* being particularly noticeable for BayesB implementations. For the 3800-SNP panel (Table 5), the respective ESS/CPU ratios were up to 80 times for *v* and up to 8 times greater for *s*^2^.

**Table 3:** **Estimates of key parameters based on BayesA and BayesB analyses of the heterogeneous stock mice dataset** (**950 SNP markers**)

	**DFMH**			**UNIMH**			**BIVMH**		
**Parameter**	**PMEAN** ^**1**^	**PMED** ^**2**^	**ESS/** **CPU** ^**3**^	**PMEAN**	**PMED**	**ESS/** **CPU**	**PMEAN**	**PMED**	**ESS/** **CPU**
**BayesA**									
_μ_	9.44E-03	9.46E-03	0.013	9.94E-03	9.88E-03	0.011	9.42E-03	9.21E-03	0.007
${\sigma}_e^2$	5.16E-04	3.99E-04	0.004	4.96E-04	3.68E-04	0.004	4.82E-04	3.50E-04	0.003
${\sigma}_c^2$	4.18E-03	4.16E-03	0.046	4.20E-03	4.18E-03	0.040	4.18E-03	4.16E-03	0.034
${\sigma}_u^2$	7.77-03	7.87E-03	0.005	7.73E-03	7.92E-03	0.004	7.78E-03	7.96E-03	0.004
*v*	20.47	7.16	0.008	46.60	7.52	0.375	37.37	7.34	0.096
*s* ^2^	2.80E-06	2.76E-06	0.004	2.84E-06	2.81E-06	0.014	2.79E-06	2.75E-06	0.011
**BayesB**									
_μ_	9.94E-03	1.02E-02	0.010	8.87E-03	8.63E-03	0.010	9.65E-03	9.70E-03	0.009
${\sigma}_e^2$	4.50E-04	3.14E-04	0.004	4.42E-04	2.81E-04	0.003	5.25E-04	4.11E-04	0.003
${\sigma}_c^2$	4.20E-03	4.18E-03	0.003	4.20E-03	4.18E-03	0.040	4.20E-03	4.18E-03	0.040
${\sigma}_u^2$	7.95E-03	8.14E-03	0.005	7.98E-03	8.20E-03	0.004	7.82E-03	7.98E-03	0.004
*v*	74.89	10.26	0.018	60.28	8.59	0.588	81.18	9.02	0.560
*s* ^2^	1.22E-05	10.4E-05	0.007	1.21E-05	1.07E-05	0.014	1.24E-05	1.08E-05	0.013
*π*	0.27	0.25	0.007	0.26	0.24	0.009	0.26	0.24	0.009

^1^Posterior means; ^2^posterior medians; ^3^effective sample size divided by total CPU time in seconds using one of three computing strategies (DFMH, UNIMH, or BIVMH) based on 80 000 MCMC cycles.

**Table 4:** **Estimates of key parameters based on BayesA and BayesB analyses of the heterogeneous stock mice dataset** (**1900 SNP markers**)

	**DFMH**			**UNIMH**			**BIVMH**		
**Parameter**	**PMEAN** ^**1**^	**PMED** ^**2**^	**ESS** **/** **CPU** ^**3**^	**PMEAN**	**PMED**	**ESS** **/** **CPU**	**PMEAN**	**PMED**	**ESS** **/** **CPU**
**BayesA**									
_μ_	8.94E-03	8.93E-03	0.009	9.55E-03	9.60E-03	0.011	9.37E-03	9.33E-03	0.023
${\sigma}_e^2$	5.43E-04	4.17E-04	0.003	5.04E-04	3.63E-04	0.003	6.31E-04	5.39E-04	0.007
${\sigma}_c^2$	4.18E-03	4.17E-03	0.035	4.18E-03	4.16E-03	0.034	4.20E-03	4.18E-03	0.109
${\sigma}_u^2$	7.37E-03	7.55E-03	0.004	7.41E-03	7.63E-03	0.004	7.19E-03	7.32E-03	0.009
*v*	18.62	6.31	0.003	54.52	6.34	0.116	36.74	6.06	0.074
*s* ^2^	1.63E-06	1.62E-06	0.002	1.70E-06	1.65E-06	0.008	1.64E-06	1.61E-06	0.020
**BayesB**									
_μ_	9.17E-03	9.17E-03	0.013	9.24E-03	9.29E-03	0.026	9.21E-03	9.18E-03	0.029
${\sigma}_e^2$	6.02E-04	5.00E-04	0.003	6.14E-04	5.15E-04	0.006	6.46E-04	5.50E-04	0.007
${\sigma}_c^2$	4.20E-03	4.18E-03	0.047	4.20E-03	4.18E-03	0.082	4.20E-03	4.18E-03	0.108
${\sigma}_u^2$	7.35E-03	7.51E-03	0.004	7.33E-03	7.47E-03	0.008	7.27E-03	7.42E-03	0.008
*v*	44.67	10.51	0.011	68.09	8.26	0.883	517.85	8.68	0.620
*s* ^2^	8.84E-06	7.66E-06	0.003	8.47E-06	7.32E-06	0.021	8.95E-06	7.83E-06	0.024
*π*	0.24	0.23	0.004	0.24	0.23	0.017	0.24	0.22	0.013

^1^Posterior means; ^2^posterior medians; ^3^effective sample size divided by total CPU time in seconds using one of three computing strategies (DFMH, UNIMH, or BIVMH) based on 80 000 MCMC cycles.

**Table 5:** **Estimates of key parameters based on BayesA and BayesB analyses of the heterogeneous stock mice dataset** (**3800 SNP markers**)

	**DFMH**			**UNIMH**			**BIVMH**		
**Parameter**	**PMEAN** ^1^	**PMED** ^**2**^	**ESS/** **CPU** ^**3**^	**PMEAN**	**PMED**	**ESS/** **CPU**	**PMEAN**	**PMED**	**ESS/** **CPU**
**BayesA**									
_μ_	8.96E-03	8.88E-03	0.008	9.43E-03	9.38E-03	0.020	8.93E-03	8.89E-03	0.023
${\sigma}_e^2$	5.43E-04	4.27E-04	0.003	6.40E-04	5.53E-04	0.006	6.47E-04	5.63E-04	0.006
${\sigma}_c^2$	4.23E-03	4.21E-03	0.029	4.26E-03	4.23E-03	0.053	4.25E-03	4.23E-03	0.088
${\sigma}_u^2$	7.06E-03	7.25E-03	0.004	6.89E-03	7.03E-03	0.006	6.87E-03	7.00E-03	0.007
*v*	3.16	2.81	0.001	3.48	2.76	0.079	3.35	2.74	0.023
*s* ^2^	4.63E-07	4.38E-07	0.002	4.38E-07	4.12E-07	0.011	4.40E-07	4.11E-07	0.017
**BayesB**									
_μ_	9.19E-03	9.32E-03	0.008	9.07E-03	9.13E-03	0.007	8.86E-03	8.83E-03	0.018
${\sigma}_e^2$	5.85E-04	4.77E-04	0.003	5.68E-04	4.52E-04	0.002	5.99E-04	5.01E-04	0.006
${\sigma}_c^2$	4.23E-03	4.21E-03	0.045	4.23E-03	4.21E-03	0.035	4.23E-03	4.21E-03	0.062
${\sigma}_u^2$	7.08E-03	7.23E-03	0.004	7.12E-03	7.30E-03	0.003	7.06E-03	7.22E-03	0.006
*v*	5081.90	3.76	0.002	7.72	3.63	0.412	5.22	3.55	0.209
*s* ^2^	4.87E-06	3.55E-06	0.002	5.38E-06	3.68E-06	0.004	4.98E-06	3.59E-06	0.011
*π*	1.99E-01	1.82E-01	0.003	1.89E-01	1.72E-01	0.004	1.93E-01	1.71E-01	0.008

^1^Posterior means; ^2^posterior medians; ^3^effective sample size divided by total CPU time in seconds using one of three computing strategies (DFMH, UNIMH, or BIVMH) based on 80 000 MCMC cycles.

In all cases, it should be noted that the posterior medians were generally in better agreement between algorithms than were the posterior means, especially for *v*. Furthermore, the posterior means were substantially higher than the corresponding medians, again especially for *v* and, in particular, for DFMH analyses based on 3800 markers (Table 5). That is, DFMH sampling for *v* periodically wandered off to extremely high values for a sustained number of cycles before revisiting more typical values (i.e. near the posterior median) during the MCMC chain; in order words, the MCMC chain mixed very poorly for DFMH.

Pairwise scatterplots of posterior means of SNP effects and GEBV between the three computing strategies are also provided in Additional file 2, with BayesA posterior mean scatterplots of SNP effects provided in Figure S9 [See Additional file 2: Figure S9], BayesB posterior mean scatterplots of SNP effects provided in Figure S10 [See Additional file 2: Figure S10], BayesA posterior mean scatterplots of GEBV provided in Figure S11 [See Additional file 2 Figure S11], and BayesB posterior mean scatterplots of GEBV provided in Figure S12 [See Additional file 2 Figure S12]. These scatterplots indicate that inferences on SNP effects and GEBV were not influenced by the three computing strategies.

## Discussion

Most researchers have not inferred key hyperparameters (i.e., *v*, *s*^2^ and *π*) that partly characterize the genetic architecture and/or marker LD or densities in BayesA or BayesB WGP models. This default action may be in part due to the high posterior correlation that exists between some of these hyperparameters, in particular, *v* and *s*^2^ [6,8]. Nevertheless, recently some researchers [28-30] have succeeded in using techniques previously presented by Yi and Xu [9] and Yang and Tempelman [3] to infer these hyperparameters in a fully Bayesian analysis; their strategies closely align with the DFMH approach that we describe in this paper.

Gianola [31] and Lehermeier *et al*. [32] have recently provided dire warnings about the arbitrary specification of these hyperparameters in parametric Bayesian WGP models such as BayesA and BayesB. Gianola [31] provided convincing analytical arguments on the large influence of the priors and the corresponding hyperparameter specifications in such models, whereas Lehermeier *et al*. [32] demonstrated with empirical results that arbitrary specification of hyperparameters can adversely impact inferences on SNP effects. In fact, they demonstrated that misspecification of *s*^2^ can lead to degradation in genomic prediction accuracy that seemed substantially greater than what we observed in our simulation study. However, it should be quickly noted that we inferred upon the remaining hyperparameters, whereas Lehermeier *et al*. [32] arbitrarily fixed *v* = 4 in BayesA and BayesB and *π* = 0.8 in BayesB. Hence, their analysis provided far less flexibility when *s*^2^ was misspecified than our analyses, since our inferences on *v* and/or *π* partly compensated for that misspecification. It seems practical and reasonable in some cases (e.g. in larger scale analyses) to infer either *v* or *s*^2^ but not necessarily both, given this compensation effect, in conjunction with the high degree of posterior correlation between the two hyperparameters. Note, for example, that the BayesD-*π* model strategy of Habier *et al*. [8], in which *v* is fixed while inferring upon *s*^2^ and *π*, might not be greatly different in its effect from fixing *s*^2^ and inferring upon *v* and *π*, as we did in our misspecification study. Our results are consistent with our previous work [3], in which more detailed explanations are provided; for example, estimates of *s*^2^ are expected to be larger with BayesB than with BayesA models since genetic variability is distributed over fewer SNPs in BayesB if *π* < < 1; for similar reasons, one would also expect smaller estimates of *s*^2^ in higher density SNP panels.

We considered two alternative sampling strategies to DFMH, each involving the use of MH, in an attempt to improve the computational efficiency of WGP analyses, as measured by the ratio ESS/CPU. Using simulation studies and empirical data analyses, we demonstrated that strategies that are more heavily based on MH and/or collapsed sampling had better computing efficiencies than the DFMH procedure first advocated by Yi and Xu [9]. Simple modifications such as sampling *s*^2^ with a MH on a collapsed FCD, or joint sampling of *s*^2^ and *v* with a bivariate MH step (BIVMH), lead to substantial improvements in ESS/CPU compared to the more common strategy based on a Gibbs step for *s*^2^ (UNIMH). We concede that our investigation is not exhaustive with respect to assessing all possible strategies to improve computing efficiency in these models; in fact, a hybrid approach may exist that combines some or all of the three presented sampling strategies that might be even computationally more efficient. Deviations of MH sampling such as Langevin-Hastings could also have been explored and assessed here, although its advantage relative to MH sampling has not been too convincing in other animal breeding models [11,12]. In work that we do not report here, we attempted to base the covariance matrix for the proposal density in BIVMH on the negative Hessian of the joint FCD of log (*v*) and log (*s*^2^). However, we determined that this matrix is positive definite generally only when *v* < 50, thereby negating its use in this way. Recently, non-MCMC (i.e. expectation-maximization) schemes have been increasingly popular. However, it is often not straightforward to estimate key hyperparameters in these implementations [5]. In any case, we encourage further development and work in this area, including the Bayesian LASSO model [33].

We also examined the components of computing efficiency; i.e., ESS and CPU/cycle in seconds separately for each parameter in both models and under all three strategies (results not reported). As anticipated, DFMH was computationally less expensive in terms of CPU/cycle compared to the proposed strategies UNIMH and BIVMH; however, the ESS were such that the values for UNIMH and BIVMH generally far exceeded those for DFMH for the same number of MCMC cycles. What was particularly worrying was how quickly the ESS/CPU measures degraded with increasing marker densities, which suggests that higher density panels, without concomitant increases in sample size, lead to analyses that require not only greater CPU/cycle but also a greater number of MCMC cycles to ensure that ESS values are sufficiently large to ensure reliable posterior inference on these hyperparameters.

We investigated the impact of these different computing strategies on GEBV and SNP effects *g*_*j*_ , as well as on fixed effects (in our case the overall mean μ). In the simulation study and in the mouse data analysis, we saved all MCMC samples from three randomly chosen *g*_*j*_ , three randomly chosen ${\displaystyle \sum_{j=1}^m{z}_{ij}}{g}_j$ (i.e., GEBV), and μ. In the simulation study, the ESS/CPU for each of these parameters always exceeded 1 for BayesA and 0.95 for BayesB, which was a substantially larger value than any of those for the hyperparameters reported in Tables 1 and 2. In the data application, we found that ESS/CPU was always several times greater for these same parameters relative to the poorest mixing parameters (i.e. ${\sigma}_u^2$ and ${\sigma}_e^2$) in Tables 3, 4 and 5, with advantages increasing with the use of UNIMH and BIVMH and with higher marker densities (results not shown). We also observed that the allele coding strategies of Stranden and Christensen [14] were essential to ensure that these computing efficiencies for GEBV, *g*_*j*_ and μ were substantially larger compared to analyses for which these allele coding strategies were not implemented (results not shown).

The joint posterior density was the same for all three algorithms; this was readily established in the simulation study but was less clear in our analysis of mice weights, where the data structure and statistical model were considerably more complex. In that situation, we noted that DFMH was particularly unstable for MCMC inference on *v*_,_ partly because MCMC draws of *v* often got stuck in high values, yet rather low probability areas of the posterior density. This situation thereby rendered inferences on *v* that were less reliable than with UNIMH and BIVMH, which facilitated good mixing throughout the joint posterior density. Since UNIMH and BIVMH are not consistently better or worse than each other in terms of computing efficiency, we recommend the use of UNIMH for its greater simplicity of implementation.

Over-specifying *s*^*2*^ appeared to have particularly deleterious effects on accuracy of genomic prediction in BayesA models, although no such effect was observed in BayesB models, which is likely due to the counteracting influence of *π*. This may be a key reason why we did not observe any differences in accuracies of GEBV in BayesB between various specifications of *s*^2^ in Figure 2. This greater robustness of BayesB to misspecification of hyperparameters could then be one reason why BayesB often outperforms BayesA in some previous reports. Note from the prior specification on ${\left\{{\sigma}_{g_j}^2\right\}}_{j=1}^m$ that $\operatorname{E}\left({\sigma}_{g_j}^2\Big|{\sigma}_{g_j}^2>0\right)=\frac{\nu {s}^2}{\nu -2};\nu >2$; that is, the average values of the draws of ${\left\{{\sigma}_{g_j}^2\right\}}_{j=1}^m$ within any one MCMC cycle will be somewhat constrained by the quantity $\frac{\nu {s}^2}{\nu -2}$ , which is not expected to be highly variable across MCMC cycles. So if *s*^2^ is understated, the estimate of *v* should decrease accordingly to compensate, so that there is a good deal of flexibility in maintaining a narrow range in $\frac{\nu {s}^2}{\nu -2}$. However, if *s*^2^ is overstated, then there is very little flexibility to accordingly bring down $\frac{\nu {s}^2}{\nu -2}$ , even with extremely large values of *v* , since $\frac{\nu {s}^2}{\nu -2}$ can never be less than *s*^2^. We hypothesize that this is the reason why understating the value of *s*^*2*^ is less serious than overstating it, at least for BayesA, as further indicated by our results, provided that the other hyperparameter *v* is inferred upon. Furthermore, specifying a very large value for *s*^2^ could create overfitting problems.

Our work on misspecification of one hyperparameter (*s*^2^) is strongly linked to the other hyperparameters (*v* and/or *π*) being inferred upon. However, all three hyperparameters are often arbitrarily specified in most BayesA or BayesB analyses. We also determined that it may be reasonable to consider specifying values for *s*^2^ for one marker density based on a previous estimate from another marker density by taking into account the inverse relationship between *s*^2^ and marker density. This might be particularly appealing, given that MCMC mixing tends to be substantially faster and to better behave for low-density panels. Hence, a reasonable strategy might be to use such analyses to extrapolate *s*^2^ specifications for higher densities, where the MCMC mixing behavior of *s*^2^ might tend to be more problematic. Or, at the very least, one could base an informative prior density for *s*^2^ based on an extrapolation from the use of a lower density panel. Perez and de los Campos [7] also discussed other strategies to specify or infer upon these hyperparameters. An even more ideal situation might be to base prior densities on posterior densities based on previous analyses of other data, since informative prior densities also facilitate better MCMC mixing.

Our simulation study and empirical study admittedly seem rather small compared to more typical analyses based on tens or even hundreds of thousands of SNPs. For example, our simulation study was based on the use of an average of *m* = 239, 598 and 2394 SNPs on a single 1 M chromosome on *n* = 1000 animals. Meuwissen and Goddard [34] demonstrated that this inferential situation should not be different from analyses based on a genome of, say, 30 chromosomes 1 M long, if all of the other specifications (number of SNPs and number of phenotypes) are scaled (i.e., by 30×) accordingly. In other words, our inferences should also characterize a situation where there are *n* = 30 000 animals with genotypes based on an average of *m* = 7170 (239×30), 17 950 (598×30) or 71 820 (2394×30) SNPs for low, medium, and high densities on a 30 M genome. Although the ESS should also be roughly the same between these two scenarios, ESS/CPU would be expected to be substantially smaller in the larger scale analyses because of larger *m* and *n*; however, this needs to be tested more extensively.

We noted that estimates of *v* progressively decreased with increasing marker densities, whether in the simulation study or within the data application. While we did not specifically investigate the effect of misspecification of *v* per se, we recognize that work has been indirectly extensively addressed in the context of BayesA (finite *v*) versus GBLUP (*v* → ∞ ) comparisons [35-38]. In fact, Nadaf *et al*. [37] demonstrated that estimated values of *v* close to 1 can lead to a greater GEBV accuracy compared to default specifications (4 ≤ *v* ≤ 5) that are typically applied in BayesA or BayesB analyses [1,7,8,32]. We suspect that the advantages of estimating *v* as opposed to setting *v* to an arbitrary value would be context-specific, as shown with *s*^2^; nevertheless, we also anticipate that misspecification of *v* would not have such dramatic consequences given the smaller differences in performance often seen between BayesA and GBLUP models.

At any rate, we provide evidence that these hyperparameters should not be arbitrarily specified in BayesA or BayesB models. We anticipate that these issues are also relevant to determine tuning parameters for various nonparametric approaches as well. However, we realize that the computational challenges may be huge for panels with marker densities that far exceed those that we considered in this paper. At the very least, some hyperparameters should be specified based on simple methods of moments-like (i.e. heritability-based) determinations [6,7] or other approximations; for example, *s*^2^ in BayesA should not be much different in magnitude from the variance component for SNP effects in a GBLUP [1] analysis; hence, a REML-like estimator could be used to provide a reasonable specification. If this is deemed to be computationally intractable relative to the marker density, then extrapolations based on analyses based on lower marker densities might be pursued, similar to those presented in this paper.

## Conclusions

In hierarchical Bayesian WGP models such as BayesA and BayesB, jointly drawing the degrees of freedom *v* and scale parameter *s*^2^ and using collapsed representations of FCD can improve MCMC efficiency for inference on all hyperparameters. Even separate univariate Metropolis-Hastings draws on *v* and *s*^2^ are substantially more efficient than using Gibbs sampling of *s*^2^. Overspecification rather than underspecification of the key hyperparameter *s*^2^ can adversely affect accuracy of GEBV under a BayesA model, even when *v* is estimated. Conversely, the BayesB model is more robust to misspecification of *s*^2^ provided that inference on *π*, the probability of association, and *v* are also inferred upon.

## Additional files

Additional file 1:
**Description of three sampling strategies.** Description: Full specification of joint posterior density and complete implementation details for all three sampling strategies (DFMH, UNIMH, and BIVMH). Additional file 2:
**Supplementary figures for simulation study (Figures S1-S5) and for analysis of mice data (Figures S6-S12).** Description: Comparison of posterior median estimates between the three sampling strategies (DFMH, UNIMH, and BIVMH) for *s* ^2^ under BayesA (Figure S1), *s* ^2^ under BayesB (Figure S2), *π* under BayesB (Figure S3), ν under BayesA (Figure S4) and ν under BayesB (Figure S5). Comparison of posterior densities of *s* ^2^ (Figure S6), ν (Figure S7), *π* (Figure S8) for the three sampling strategies. Scatterplot comparisons of posterior means of SNP effects between the three sampling strategies under BayesA (Figure S9) and BayesB (Figure S10) based on mice data. Scatterplot comparisons of posterior means of genomic breeding values between the three sampling strategies under BayesA (Figure S11) and BayesB (Figure S12) based on mice data.
