# Supplementary material for: Improving the computational efficiency of fully Bayes inference and assessing the effect of misspecification of hyperparameters in whole-genome prediction models
Source: Genet Sel Evol. 2015 Mar 7;47(1):13. doi: 10.1186/s12711-015-0092-x (PMC4351701; doi:10.1186/s12711-015-0092-x)
Supplement: Additional file 1: — Description of three sampling strategies. Description: Full specification of joint posterior density and complete implementation details for all three sampling strategies (DFMH, UNIMH, and BIVMH). [file 12711_2015_92_MOESM1_ESM.pdf]

Additional File 1

Joint posterior density

The joint posterior density of all unknowns under DFMH can be written as follows:

$$p\left(\boldsymbol{\beta}, g_1, g_2, \ldots g_m, \sigma^2_{g_1}, \sigma^2_{g_1}, \ldots, \sigma^2_{g_m}, \sigma^2_e, s^2, \nu, \pi \mid \mathbf{y}\right) \propto$$

$$\left(\prod_{i=1}^{n} p\left(y_i \mid \boldsymbol{\beta}, g_1, g_2, \ldots, g_m, \sigma^2_e\right)\right)\left(\prod_{j=1}^{m} p\left(g_j \mid \sigma^2_{g_j}\right) p\left(\sigma^2_{g_j} \mid \nu, s^2, \pi\right)\right) p\left(s^2_g \mid \alpha_s, \beta_s\right) p\left(\nu_g\right) p\left(\pi \mid \alpha_\pi, \beta_\pi\right) p\left(\boldsymbol{\beta}\right) p\left(\sigma^2_e\right)$$

where

$$p\left(y_i \mid \boldsymbol{\beta}, g_1, g_2, \ldots, g_m, \sigma^2_e\right) = \left(2\pi\sigma^2_e\right)^{-n/2} \exp\left(-\frac{1}{2\sigma^2_e}\sum_{i=1}^{n}\left(y_i - \mathbf{x}'_i\boldsymbol{\beta} - \sum_{j=1}^{m} z_{ij} g_g\right)^2\right),$$

$$p\left(g_j \mid \sigma^2_{g_j}\right) = \left(2\pi\sigma^2_{g_i}\right)^{-1/2} \exp\left(-\frac{1}{2\sigma^2_{g_j}} g_j^{\,2}\right),$$

$$p\left(\sigma^2_{g_j} \mid \nu, s^2, \pi\right) = \begin{cases} \dfrac{\left(\dfrac{\nu s^2}{2}\right)^{\frac{\nu}{2}}}{\Gamma\left(\dfrac{\nu}{2}\right)} \sigma^{2^{-\left(\frac{\nu}{2}+1\right)}}_{g_i} e^{-\frac{\nu s^2}{2\sigma^2_{g_i}}} & \text{with probability } \pi \\ 0 & \text{with probability } (1-\pi) \end{cases},$$

$$p\left(s^2_g \mid \alpha_s, \beta_s\right) = \frac{(\beta_s)^{\alpha_s}}{\Gamma\left(\alpha_s\right)}\left(s^2\right)^{\alpha_s - 1} e^{-\beta_s s^2},$$

$$p\left(\nu_g\right) = \frac{1}{\left(1+\nu_g\right)^2},$$

$$p\left(\pi \mid \alpha_\pi, \beta_\pi\right) \propto \pi^{\alpha_\pi - 1}\left(1-\pi\right)^{\beta_\pi - 1},$$

and $p(\boldsymbol{\beta})$ and $p\left(\sigma_e^2\right)$ can be arbitrarily specified to be informative or diffuse priors with different functional distributional forms.

For UNIMH and BIVMH, the joint posterior density is further marginalized to integrate out the uncertainty on $\sigma_{g_1}^2, \sigma_{g_1}^2, ..., \sigma_{g_m}^2$; i.e.

$$p\left(\boldsymbol{\beta}, g_1, g_2, ...g_m, \sigma_e^2, s^2, \nu, \pi \mid \mathbf{y}\right) \propto$$
$$\left(\prod_{i=1}^{n} p\left(y_i \mid \boldsymbol{\beta}, g_1, g_2, ..., g_m, \sigma_e^2\right)\right)\left(\prod_{j=1}^{m} p\left(g_j \mid \nu, s^2, \pi\right)\right) p\left(s_g^2 \mid \alpha_s, \beta_s\right) p\left(\nu_g\right) p\left(\pi \mid \alpha_\pi, \beta_\pi\right) p(\boldsymbol{\beta}) p\left(\sigma_e^2\right)$$

where

$$p\left(g_j \mid \nu, s^2, \pi\right) = \int_{\sigma_{g_j}^2} p\left(g_j \mid \sigma_{g_j}^2\right) p\left(\sigma_{g_j}^2 \mid \nu, s^2, \pi\right) d\sigma_{g_j}^2$$
$$= \begin{cases} \dfrac{\Gamma\left(\dfrac{\nu+1}{2}\right)}{\Gamma\left(\dfrac{\nu}{2}\right)}\left(\dfrac{1}{\pi \nu s^2}\right)^{1/2}\left[1+\dfrac{g_j^2}{\nu s^2}\right]^{-\frac{\nu+1}{2}} & \text{with probability } \pi \\ 0 & \text{with probability } (1-\pi) \end{cases}$$

i.e., a mixture of a scaled Student *t* distribution with probability π and a point mass at zero of probability (1-π).

Description of the three sampling strategies

Sampling strategy for DFMH

Sampling the degrees of freedom parameter for the random SNP effects: We used a proper prior $p(\nu) \propto (\nu+1)^{-2}$ which corresponds to a Uniform(0,1) prior on $(\nu+1)^{-1}$. The full conditional density (FCD) for ν can be written as follows:

$$p(\nu \mid ELSE) \propto \left(\prod_{j=1}^{m} I\left(\sigma_{g_j}^2 > 0\right) p\left(\sigma_{g_j}^2 \mid \nu, s^2\right) \right) p(\nu)$$

$$= \left(\prod_{j=1}^{m} I\left(\sigma_{g_j}^2 > 0\right) \frac{\left(\frac{\nu s^2}{2}\right)^{\frac{\nu}{2}}}{\Gamma\left(\frac{\nu}{2}\right)} \sigma_{g_j}^{2^{-\left(\frac{\nu}{2}+1\right)}} e^{-\frac{\nu s^2}{2\sigma_{g_j}^2}} \right) \frac{1}{(1+\nu)^2}$$

where $I(.)$ is the indicator variable such that if the condition inside (.) is true, $I(.) = 1$, otherwise $I(.) = 0$. As this FCD is not recognizable, we propose a random walk normal Metropolis Hastings (MH) step on $\xi = \log(\nu)$. Noting that the Jacobian from ν to ξ is $\exp(\xi)$, the corresponding FCD for ξ is as follows:

$$p(\xi \mid ELSE)$$

$$\propto \left(\frac{\left(exp(\xi)s^2/2\right)^{exp(\xi)/2}}{\Gamma\left(exp(\xi)/2\right)} \right)^{m_1} \left(\prod_{j=1}^{m} I\left(\sigma_{g_j}^2 > 0\right) \sigma_{g_j}^{2^{-\left(\frac{exp(\xi)}{2}+1\right)}} e^{-\frac{exp(\xi)s^2}{2\sigma_{g_j}^2}} \right) \frac{1}{\left(1+exp(\xi)\right)^2} exp(\xi)$$

where $m_1 = \sum_{j=1}^{m} I\left(\sigma_{g_j}^2 > 0\right)$. Hence

$$\log p\left(\xi \mid ELSE\right)$$
$$= m_1\left(\frac{exp(\xi)}{2}\left(\xi + \log(s^2) - \log(2)\right) - \log\Gamma\left(\frac{exp(\xi)}{2}\right)\right) +$$
$$\sum_{j=1}^{m} I\left(\sigma_{g_j}^2 > 0\right)\left(-\left(\frac{exp(\xi)}{2}+1\right)\log(\sigma_{g_j}^2) - \frac{exp(\xi)s^2}{2\sigma_{g_j}^2}\right) - 2\log(1+exp(\xi)) + \xi$$

Suppose the value of ξ in the current cycle *i* is $\xi^{[i]}$, the random walk proposal for $\xi^{[i+1]}$ in the next MCMC cycle is drawn from a Gaussian distribution:

$$p\left(\xi*\right) = \frac{1}{\sqrt{2\pi}c_{\mathbf{v}}}\exp\left(\frac{-\left(\xi * - \xi^{[i]}\right)^2}{2c_{\mathbf{v}}^2}\right)$$

This is equivalent to generating a random variable, say δ from N(0, c_v^2) and adding it to $\xi^{[i]}$ to propose $\xi^* = \xi^{[i]} + \delta$. The MH acceptance ratio is determined to be $\alpha = \frac{p\left(\xi* \mid ELSE\right)}{p\left(\xi^{[i]} \mid ELSE\right)}$. For numerical stability, we evaluated this ratio as:

$$\alpha = \exp\left(\log p\left(\xi* \mid ELSE\right) - \log p\left(\xi^{[i]} \mid ELSE\right)\right)$$

To implement this MH sampling strategy, we generate u from a Uniform(0,1) distribution. If $\alpha > 1$, accept $\xi^{[i+1]} = \xi *$. If $u < \alpha$ then set $\xi^{[i+1]} = \xi *$. Otherwise if $u > \alpha$, then set $\xi^{[i+1]} = \xi^{[i]}$.

The following tuning procedure adapted from Muller [1] is to determine c_v^2:

1) For every 10 cycles, if the rate of acceptance is greater than 80%, increase c_v^2 by a factor of 1.2.

2) For every 10 cycles if the rate of acceptance is less than 20%, decrease c_v^2 by a factor of 0.7.

3) After burn-in, keep c_v^2 constant and monitor subsequent acceptance rates to ensure that they fall within 25 to 75%.

Sampling the scale parameter for the random SNP effects: Borrowing results from Yi and Xu [2], the FCD for s^2 based on the specification of a conjugate prior $p\left(s^2 \mid \alpha_s, \beta_s\right)$ = Gamma $\left(\alpha_s, \beta_s\right)$ can be written as follows:

$$p\left(s^2 \mid ELSE\right) \propto \left(\prod_{j=1}^{m} I\left(\sigma_{g_j}^2 > 0\right) p\left(\sigma_{g_j}^2 \mid \nu, s^2\right)\right) p\left(s^2 \mid \alpha_s, \beta_s\right)$$

$$= \left(\prod_{j=1}^{m} I\left(\sigma_{g_j}^2 > 0\right) \frac{\left(\frac{s^2 \nu}{2}\right)^{\frac{\nu}{2}}}{\Gamma\left(\frac{\nu}{2}\right)} \sigma_{g_j}^{2\,-\left(\frac{\nu}{2}+1\right)} e^{-\frac{s^2\nu}{2\sigma_{g_j}^2}}\right) \frac{\left(\beta_s\right)^{\alpha_s}}{\Gamma\left(\alpha_s\right)} \left(s^2\right)^{\alpha_s - 1} e^{-\beta_s s^2}$$

$$\propto \left(s^2\right)^{\alpha_s + \frac{m_1 \nu}{2} - 1} \exp\left(-s^2 \left(\frac{\nu}{2} \sum_{j=1}^{m} I\left(\sigma_{g_j}^2 > 0\right) \sigma_{g_j}^{-2} + \beta_s\right)\right)$$

i.e., a Gamma distribution with parameters $\alpha_s + \frac{m_1 \nu}{2}$ and $\frac{\nu}{2} \sum_{j=1}^{m} I\left(\sigma_{g_j}^2 > 0\right) \sigma_{g_j}^{-2} + \beta_s$.

Sampling strategy for UNIMH

Sampling the degrees of freedom parameter for the random SNP effects: The "collapsed" FCD for sampling ν (after integrating out $\left\{\sigma^2_{g_j}\right\}_{j=1}^{m}$ from the FCD for ν as specified in DFMH above) is as follows:

$$p(\nu \mid ELSE) \propto \left(\prod_{j=1}^{m} I(g_j \neq 0)\, p(g_j \mid \nu_g, s_g^2)\right) p(\nu)$$

$$= \left[\prod_{j=1}^{m} I(g_j \neq 0) \frac{\Gamma\left(\frac{\nu+1}{2}\right)}{\Gamma\left(\frac{\nu}{2}\right)} \left(\frac{1}{\pi \nu s^2}\right)^{1/2} \left[1+\frac{g_j^2}{\nu s^2}\right]^{-\frac{\nu+1}{2}}\right] \frac{1}{(1+\nu)^2}$$

As this FCD is not recognizable, we specify a random walk MH step on $\xi = \log(\nu)$. Note that the Jacobian from ν to ξ is $\exp(\xi)$. The corresponding FCD for ξ is as follows:

$$p(\xi \mid ELSE)$$

$$\propto \left(\frac{\Gamma((exp(\xi)+1)/2)}{\Gamma(exp(\xi)/2)}\right)^{m_1} \left(\frac{1}{\pi exp(\xi) s^2}\right)^{m_1/2} \left(\prod_{j=1}^{m} I(g_j \neq 0) \left[1+\frac{g_j^2}{exp(\xi) s^2}\right]^{-\frac{exp(\xi)+1}{2}}\right)$$

$$\frac{1}{(1+exp(\xi))^2} exp(\xi)$$

where $m_1 = \sum_{j=1}^{m} I(g_j \neq 0)$. Hence

$$\log p\left(\xi \mid ELSE\right)$$
$$= m_1\left(\log\Gamma\left(\frac{exp(\xi)+1}{2}\right) - \log\Gamma\left(\frac{exp(\xi)}{2}\right) + \frac{1}{2}\log\left(\frac{1}{\pi exp(\xi)s^2}\right)\right) +$$
$$\sum_{j=1}^{m} I\left(g_j \neq 0\right)\left(-\left(\frac{exp(\xi)+1}{2}\right)\log(1+\frac{g_j^2}{exp(\xi)s^2})\right) - 2\log(1+exp(\xi)) + \xi$$

Suppose the value of ξ in the current cycle *i* is $\xi^{[i]}$. We propose a random walk sample for $\xi^{[i+1]}$ in the next cycle using a Gaussian proposal distribution:

$$p\left(\xi^*\right) = \frac{1}{\sqrt{2\pi}c_{\mathbf{v}}}\exp\left(\frac{-\left(\xi^* - \xi^{[i]}\right)^2}{2c_{\mathbf{v}}^2}\right)$$

That is equivalent to generating a random variable, say δ from N(0, c_v^2) and adding it to $\xi^{[i]}$ to propose $\xi^* = \xi^{[i]} + \delta$. To determine the MH acceptance ratio $\alpha = \frac{p\left(\xi^* \mid ELSE\right)}{p\left(\xi^{[i]} \mid ELSE\right)}$, we evaluated this ratio in a numerically stable manner as:

$$\alpha = \exp\left(\log p\left(\xi^* \mid ELSE\right) - \log p\left(\xi^{[i]} \mid ELSE\right)\right)$$

To implement this MH sampling strategy, we first generated u from a Uniform(0,1) distribution. If $\alpha > 1$ or $u < \alpha$ then set $\xi^{[i+1]} = \xi^*$ otherwise set $\xi^{[i+1]} = \xi^*$. The same tuning procedure from Muller [1] as described in DFMH for c_v^2 was used here.

Sampling the scale parameter for the random SNP effects: The FCD for sampling s^2 is as follows:

$$p\left(s^2 \mid ELSE\right) \propto \left(\prod_{j=1}^{m} I\left(g_j \neq 0\right) p\left(g_j \mid \nu, s^2\right) \right) p\left(s^2 \mid \alpha_s, \beta_s\right)$$

$$= \left(\prod_{j=1}^{m} I\left(g_j \neq 0\right) \frac{\Gamma\left(\frac{\nu+1}{2}\right)}{\Gamma\left(\frac{\nu}{2}\right)} \left(\frac{1}{\pi \nu s^2}\right)^{1/2} \left[1 + \frac{g_j^2}{\nu s^2}\right]^{-\frac{\nu+1}{2}} \right) \frac{\left(\beta_s\right)^{\alpha_s}}{\Gamma\left(\alpha_s\right)} \left(s^2\right)^{\alpha_s - 1} e^{-\beta_s s^2}$$

Even though this FCD is recognizable, we propose a random walk MH step on $\psi = \log(s^2)$. Note that the Jacobian from s^2 to ψ is $\exp(\psi)$. The corresponding FCD for ψ is as follows:

$$p\left(\psi \mid ELSE\right)$$

$$\left(\prod_{j=1}^{m} I\left(g_j \neq 0\right) \frac{\Gamma\left(\frac{\nu+1}{2}\right)}{\Gamma\left(\frac{\nu}{2}\right)} \left(\frac{1}{\pi \nu s^2}\right)^{1/2} \left[1 + \frac{g_j^2}{\nu s^2}\right]^{-\frac{\nu+1}{2}} \right) \frac{\left(\beta_s\right)^{\alpha_s}}{\Gamma\left(\alpha_s\right)} \left(s^2\right)^{\alpha_s - 1} e^{-\beta_s s^2}$$

$$\propto \left(\frac{\Gamma\left((\nu+1)/2\right)}{\Gamma\left(\nu/2\right)} \right)^{m_1} \left(\frac{1}{\pi \nu exp(\psi)} \right)^{m_1/2} \left(\prod_{j=1}^{m} I\left(g_j \neq 0\right) \left[1 + \frac{g_j^2}{\nu exp(\psi)}\right]^{-\frac{\nu+1}{2}} \right)$$

$$\left(exp(\psi)\right)^{\alpha_s - 1} e^{-\beta_s exp(\psi)} exp(\psi)$$

where $m_1 = \sum_{j=1}^{m} I\left(g_j \neq 0\right)$. Hence

$$\log p\left(\psi \mid ELSE\right)$$

$$= m_1 \left(\log\Gamma\left(\frac{\nu+1}{2}\right) - \log\Gamma\left(\frac{\nu}{2}\right) + \frac{1}{2}\log\left(\frac{1}{\pi \nu exp(\psi)}\right) \right) +$$

$$\sum_{j=1}^{m} I\left(g_j \neq 0\right) \left(-\left(\frac{\nu+1}{2}\right) \log\left(1 + \frac{g_j^2}{\nu exp(\psi)}\right) \right) + \left(\alpha_s - 1\right)\psi - \beta_s exp(\psi) + \psi$$

Suppose the value of ψ in the current cycle i is $\psi^{[i]}$. We propose a random walk value for $\psi^{[i+1]}$ in the next cycle from a Gaussian distribution:

$$p(\psi^*) = \frac{1}{\sqrt{2\pi}c_s}\exp\left(\frac{-\left(\psi^* - \psi^{[i]}\right)^2}{2c_s^2}\right)$$

That is equivalent to generate a random variable, say δ from $N(0, c_s^2)$ and adding it to $\psi^{[i]}$ to propose $\psi^* = \psi^{[i]} + \delta$. To determine the acceptance ratio $\alpha = \frac{p(\psi^* \mid ELSE)}{p(\psi^{[i]} \mid ELSE)}$ in a numerically stable manner, we evaluated this ratio as:

$$\alpha = \exp\left(\log p(\psi^* \mid ELSE) - \log p\left(\psi^{[i]} \mid ELSE\right)\right)$$

To implement this MH sampling strategy, we first generated u from a Uniform(0,1) distribution. If $\alpha > 1$ or $u < \alpha$, set $\psi^{[i+1]} = \psi^*$. Otherwise, if $u > \alpha$ then set $\psi^{[i+1]} = \psi^{[i]}$. The same tuning procedure from Muller [1] described previously is used to tune c_s^2:

Sampling strategy for BIVMH

Sampling the degrees of freedom and scale parameters for the random SNP effects:

We divided burn-in into four stages with equal length:

Stage 1: Sample $\log(\nu)$ and $\log(s^2)$ using UNIMH (see sampling strategy 2) with fine-tuning procedure on c_ν^2 and c_s^2 , which are also the variances for the two separate Gaussian proposal densities;

Stage 2: Sample $\log(\nu)$ and $\log(s^2)$ using UNIMH with fixing c_ν^2 and c_s^2 to the values tuned from the last cycle in stage 1 and compute correlation r between samples of $\log(\nu)$ and $\log(s^2)$ within stage 2;

Stage 3: Jointly sample $\log(\nu)$ and $\log(s^2)$ using a bivariate Gaussian proposal density with variances c_ν^2 and c_s^2 based on those tuned at the end of Stage 1 and a covariance based on the correlation computed from Stage 2. The joint FCD for ν and s^2 (based on a "collapsed specification that integrates out $\left\{\sigma_{g_j}^2\right\}_{j=1}^m$ from the FCD) is as follows:

$$p\left(\nu,s^2 \mid ELSE\right) \propto \left(\prod_{j=1}^m I\left(g_j \neq 0\right) p\left(g_j \mid \nu,s^2\right)\right) p\left(\nu\right) p\left(s^2 \mid \alpha_s,\beta_s\right)$$

$$= \left(\prod_{j=1}^m I\left(g_j \neq 0\right) \frac{\Gamma\left(\frac{\nu+1}{2}\right)}{\Gamma\left(\frac{\nu}{2}\right)} \left(\frac{1}{\pi \nu s^2}\right)^{1/2} \left[1+\frac{g_j^2}{\nu s^2}\right]^{-\frac{\nu+1}{2}}\right) \frac{1}{\left(1+\nu\right)^2} \frac{\left(\beta_s\right)^{\alpha_s}}{\Gamma\left(\alpha_s\right)} \left(s^2\right)^{\alpha_s-1} e^{-\beta_s s^2}$$

As this density is not recognizable, we could use a random walk normal MH step on $\xi = \log(\nu)$ and $\psi = \log(s^2)$. Note that the Jacobian from ν to ξ is $\exp(\xi)$ whereas the Jacobian from s^2 to ψ is $\exp(\psi)$. The corresponding FCD for ξ and ψ is as follows:

$$p\left(\xi,\psi \mid ELSE\right)$$

$$= \left(\frac{\Gamma\left((exp(\xi)+1)/2\right)}{\Gamma\left(exp(\xi)/2\right)}\right)^{m_1} \left(\frac{1}{\pi exp(\xi) exp(\psi)}\right)^{m_1/2} \left(\prod_{j=1}^m I\left(g_j \neq 0\right) \left[1+\frac{g_j^2}{exp(\xi)exp(\psi)}\right]^{-\frac{exp(\xi)+1}{2}}\right)$$

$$\frac{1}{\left(1+exp(\xi)\right)^2} \left(exp(\psi)\right)^{\alpha_s-1} e^{-\beta_s exp(\psi)} exp(\psi) exp(\xi)$$

Where $m_1 = \sum_{j=1}^{m} I\left(g_j \neq 0\right)$, hence

$$\begin{aligned}
&\log p\left(\xi,\psi \mid ELSE\right) \\
&= m_1\left(\log\Gamma\left(\frac{exp(\xi)+1}{2}\right) - \log\Gamma\left(\frac{exp(\xi)}{2}\right) + \frac{1}{2}\left(\frac{1}{\pi exp(\xi) exp(\psi)}\right)\right) + \\
&\sum_{j=1}^{m} I\left(g_j \neq 0\right)\left(-\left(\frac{exp(\xi)+1}{2}\right)\log(1+\frac{g_j^2}{exp(\xi)exp(\psi)})\right) - \\
&2\log(1+exp(\xi)) + \left(\alpha_s - 1\right)\psi - \beta_s exp(\psi) + \psi + \xi
\end{aligned}$$

Suppose the value of $\boldsymbol{\eta} = \left[\xi,\psi\right]'$ in the current cycle *i* is $\boldsymbol{\eta}^{[i]}$. We propose a random walk for $\boldsymbol{\eta}^{[i+1]}$ in the next cycle from a bivariate Gaussian distribution:

$$p\left(\boldsymbol{\eta}^*\right) = \frac{1}{2\pi\left|c_{\boldsymbol{\eta}}^2\boldsymbol{\Sigma}\right|^{1/2}} \exp\left(-\left(\boldsymbol{\eta}^* - \boldsymbol{\eta}^{[i]}\right)'\left(c_{\boldsymbol{\eta}}^2\boldsymbol{\Sigma}\right)^{-1}\left(\boldsymbol{\eta}^* - \boldsymbol{\eta}^{[i]}\right)\right)$$

That is equivalent to generate a random variable, say $\boldsymbol{\delta}$ from **N**(**0**, $c_{\boldsymbol{\eta}}^2\boldsymbol{\Sigma}$) and add it to $\boldsymbol{\eta}^{[i]}$ to propose $\boldsymbol{\eta}^* = \boldsymbol{\eta}^{[i]} + \boldsymbol{\delta}$, where $\boldsymbol{\Sigma} = \begin{bmatrix} c_v^2 & r\sqrt{c_v^2 c_s^2} \\ r\sqrt{c_v^2 c_s^2} & c_s^2 \end{bmatrix}$, c_v^2 and c_s^2 were determined after tuning at the end of Stage 1 and the correlation r between samples of $\log(\nu)$ and $\log(s^2)$ is based on samples drawn during Stage 2.

To determine the acceptance ratio $\alpha = \frac{p\left(\boldsymbol{\eta}^* \mid ELSE\right)}{p\left(\boldsymbol{\eta}^{[i]} \mid ELSE\right)}$, we evaluated this ratio in a numerically stable manner as:

$$\alpha = \exp\left(\log p\left(\boldsymbol{\eta}^* \mid ELSE\right) - \log p\left(\boldsymbol{\eta}^{[i]} \mid ELSE\right)\right)$$

To implement this Metropolis sampling strategy, we first generated u from a Uniform(0,1) distribution. If $\alpha > 1$ or $u < \alpha$ then set $\boldsymbol{\eta}^{[i+1]} = \boldsymbol{\eta}^*$. Otherwise, if $u > \alpha$, then set $\boldsymbol{\eta}^{[i+1]} = \boldsymbol{\eta}^{[i]}$. The Muller [1] tuning procedure was adapted as follows to determine c_{η}^2.

1) For the last 10 cycles, the rate of acceptance is greater than 60%, increase c_{η}^2 by a factor of 1.2.

2) For the last 10 cycles, the rate of acceptance is less than 10%, decrease c_{η}^2 by a factor of 0.7.

3) After the burn-in, keep c_{η}^2 constant and monitor subsequent acceptance rates to ensure that they fall within 25 to 75%.

Stage 4: Jointly sample $\log(\nu)$ and $\log(s^2)$ using a bivariate Gaussian proposal density with fixing value of c_{η}^2 at the end of Stage 3. After burn-in, save all samples on ν and s^2 using MH with the bivariate Gaussian proposal density.

REFERENCES

[1] Muller P., A generic approach to posterior integration and Gibbs sampling. Technical Report 91-09 [on line] (1991) http://www.stat.purdue.edu/research/technical_reports/1991-tr.html [consulted June 10 2014].

[2] Yi N., Xu S., Bayesian Lasso for quantitative trait loci mapping, Genetics. 179 (2008) 1045 - 1055.
