# Supplementary material for: Improving the computational efficiency of fully Bayes inference and assessing the effect of misspecification of hyperparameters in whole-genome prediction models
Source: Genet Sel Evol. 2015 Mar 7;47(1):13. doi: 10.1186/s12711-015-0092-x (PMC4351701; doi:10.1186/s12711-015-0092-x)
Supplement: Additional file 2: — Supplementary figures for simulation study (Figures S1-S5) and for analysis of mice data (Figures S6-S12). Description: Comparison of posterior median estimates between the three sampling strategies (DFMH, UNIMH, and BIVMH) for s 2 under BayesA (Figure S1), s 2 under BayesB (Figure S2), π under BayesB (Figure S3), ν under BayesA (Figure S4) and ν under BayesB (Figure S5). Comparison of posterior densities of s 2 (Figure S6), ν (Figure S7), π (Figure S8) for the three sampling strategies. Scatterplot comparisons of posterior means of SNP effects between the three sampling strategies under BayesA (Figure S9) and BayesB (Figure S10) based on mice data. Scatterplot comparisons of posterior means of genomic breeding values between the three sampling strategies under BayesA (Figure S11) and BayesB (Figure S12) based on mice data. [file 12711_2015_92_MOESM2_ESM.pdf]

Additional File 2

Supplementary figures and tables

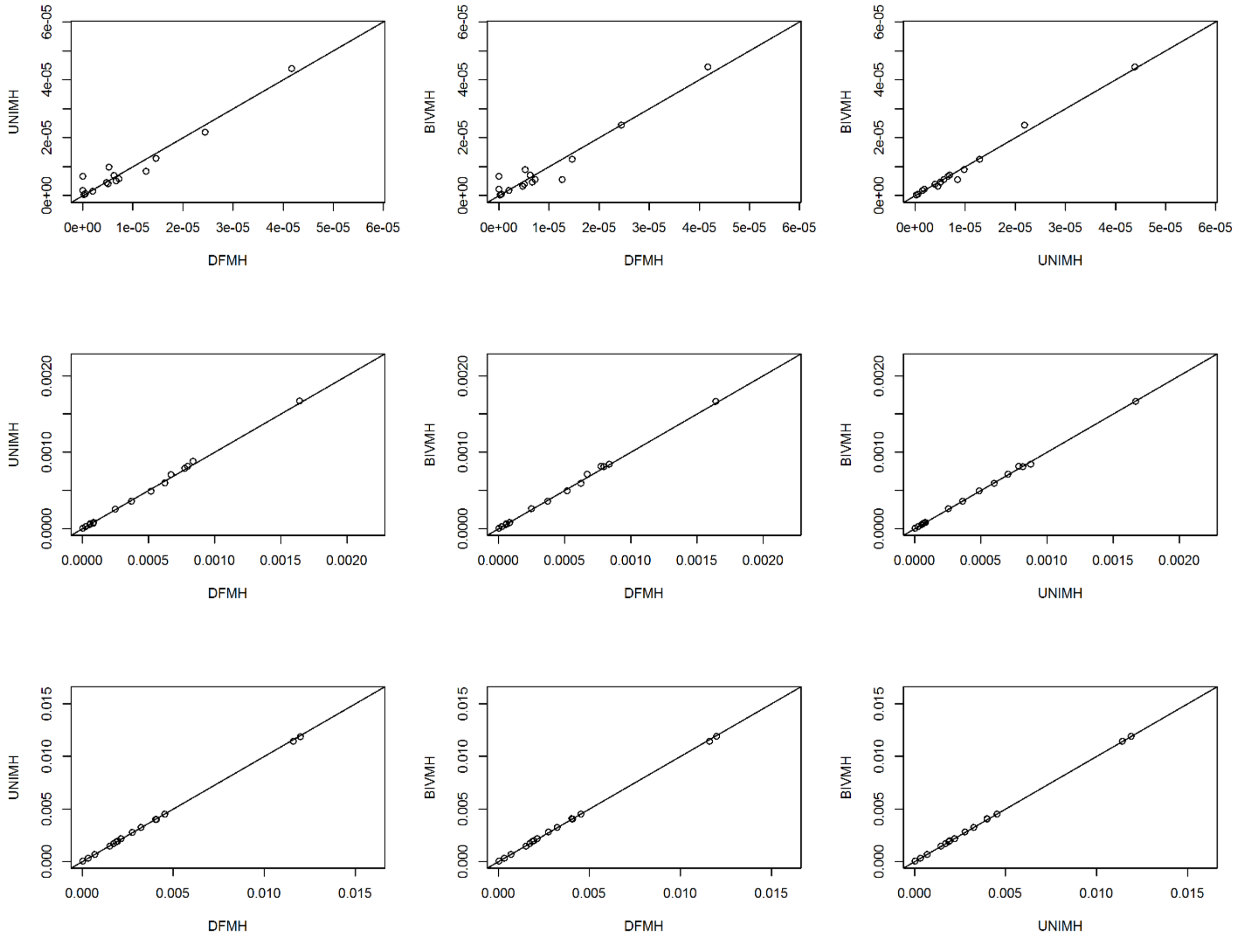

Figure S1: A comparison of posterior median estimates of s^2 across 15 replicates between DFMH, UNIMH, and BIVMH computing strategies under a BayesA model at three different LD levels of $r^2 = 0.17$ (bottom row), $r^2 = 0.24$(middle row) and $r^2 = 0.32$ (top row).

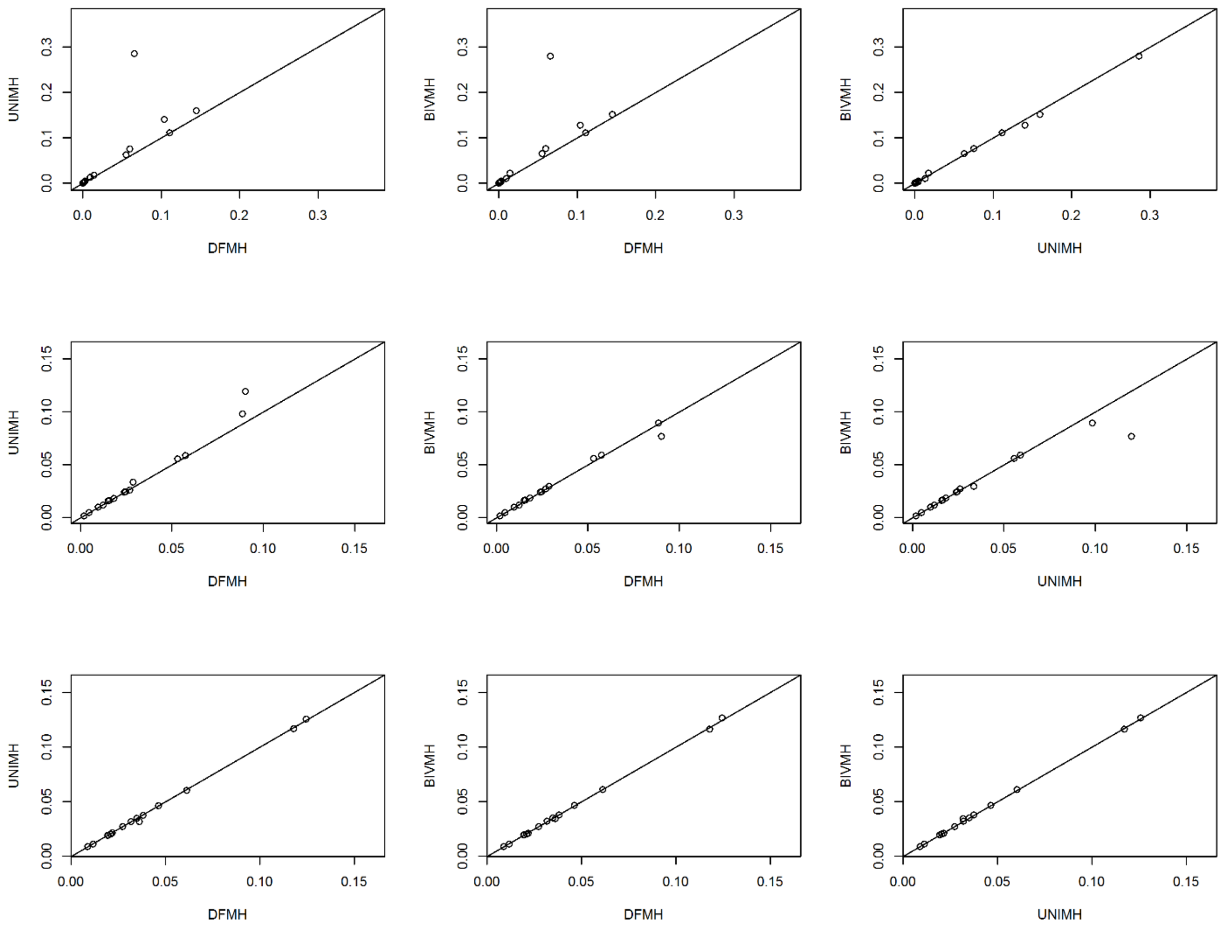

Figure S2: A comparison of posterior median estimates of s^2 across 15 replicates between DFMH, UNIMH, and BIVMH computing strategies under a BayesB model at three different LD levels of $r^2 = 0.17$ (bottom row), $r^2 = 0.24$(middle row) and $r^2 = 0.32$ (top row).

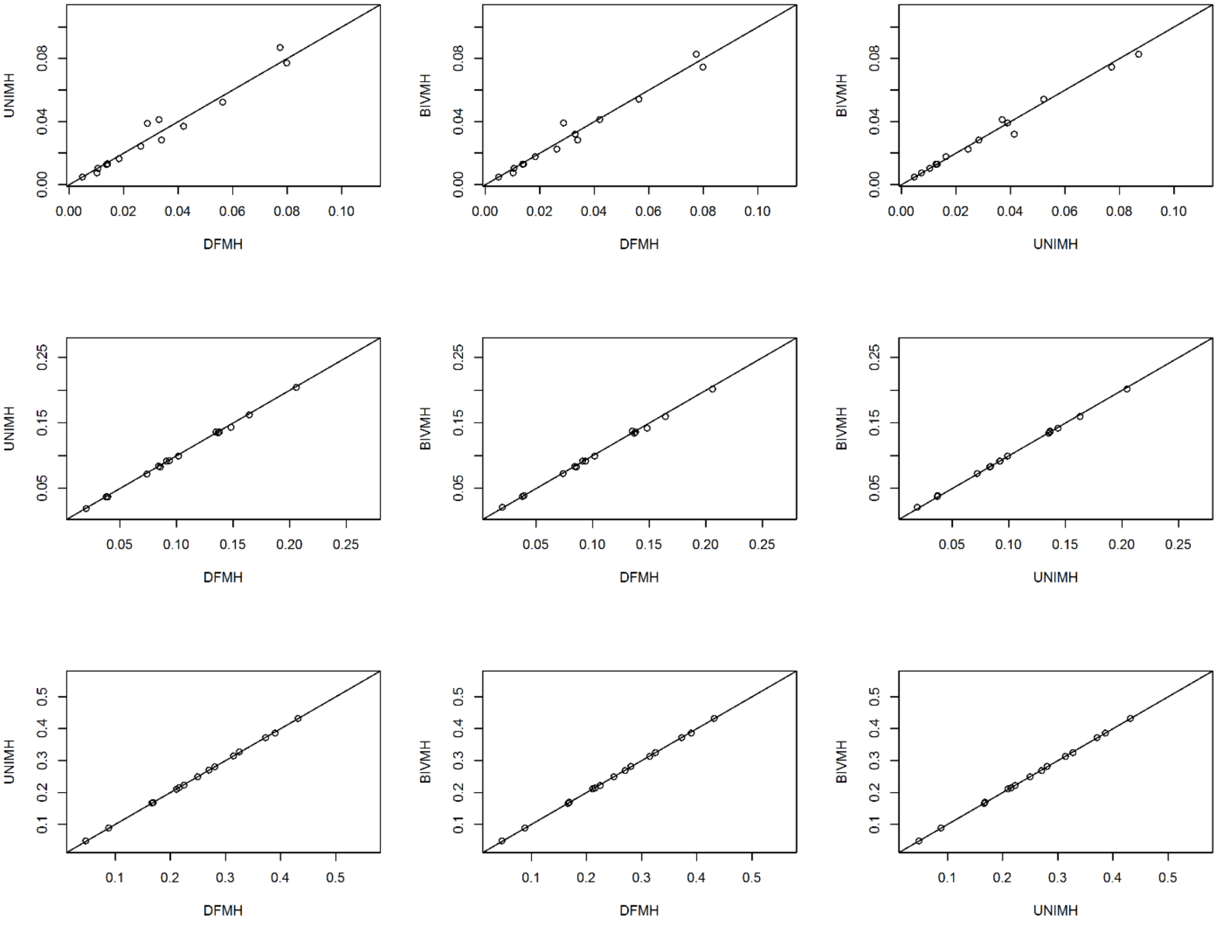

Figure S3: A comparison of posterior median estimates of π across 15 replicates between DFMH, UNIMH, and BIVMH computing strategies under a BayesB model at three different LD levels of $r^2 = 0.17$ (bottom row), $r^2 = 0.24$(middle row) and $r^2 = 0.32$ (top row).

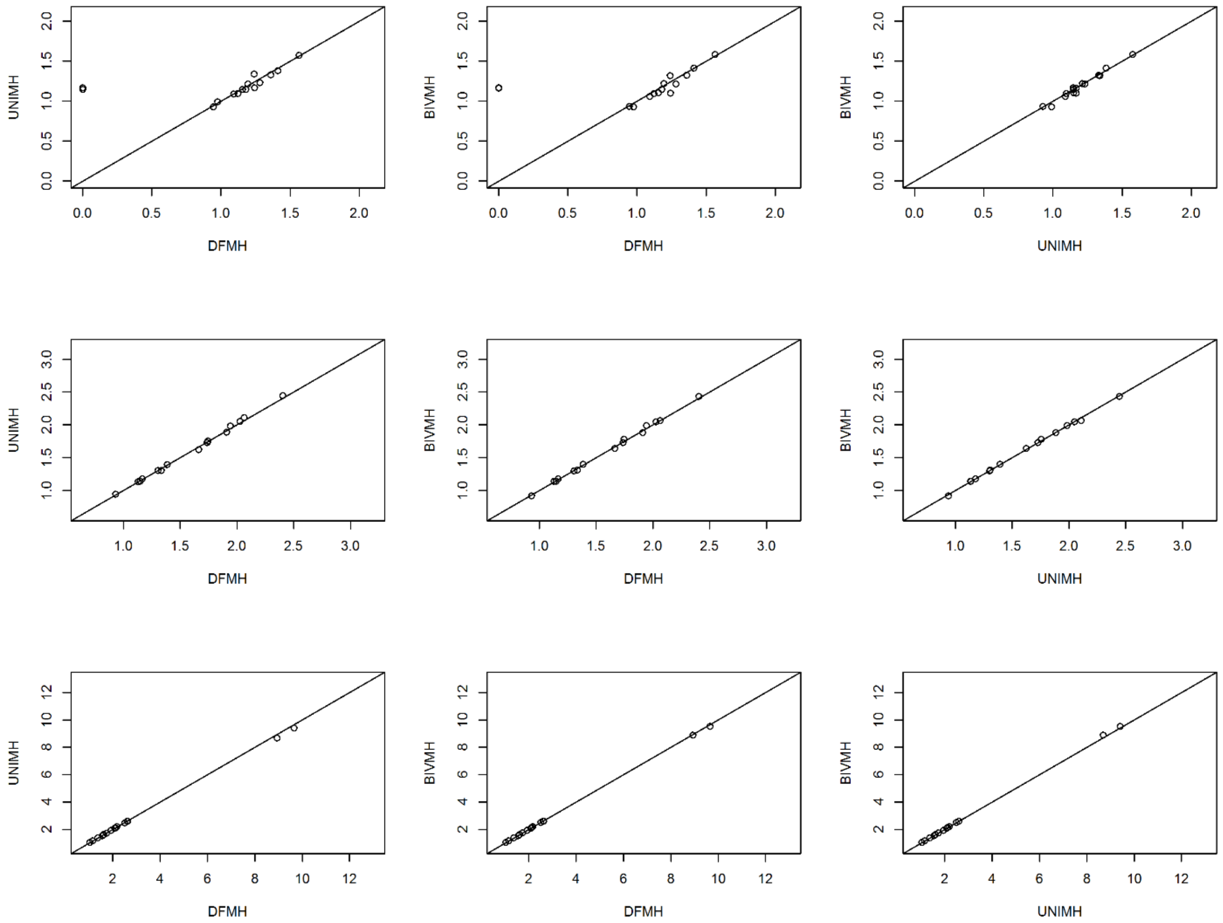

Figure S4: A comparison of posterior median estimates of ν across 15 replicates between DFMH, UNIMH, and BIVMH computing strategies under a BayesA model at three different LD levels of r^2 = 0.17 (bottom row), r^2 = 0.24(middle row) and r^2 = 0.32 (top row).

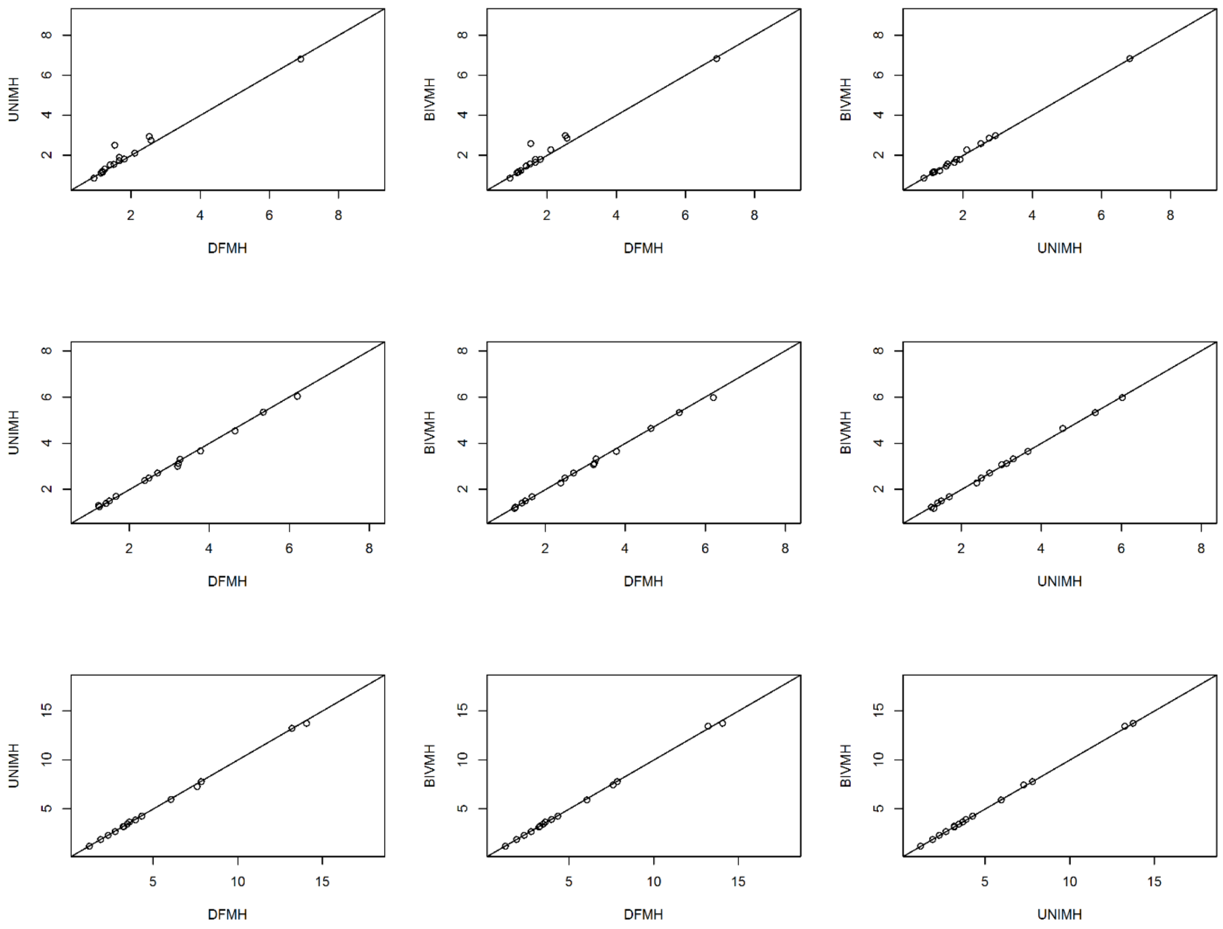

Figure S5: A comparison of posterior median estimates of ν across 15 replicates between DFMH, UNIMH, and BIVMH computing strategies under a BayesB model at three different LD levels of r^2 = 0.17 (bottom row), r^2 = 0.24(middle row) and r^2 = 0.32 (top row).

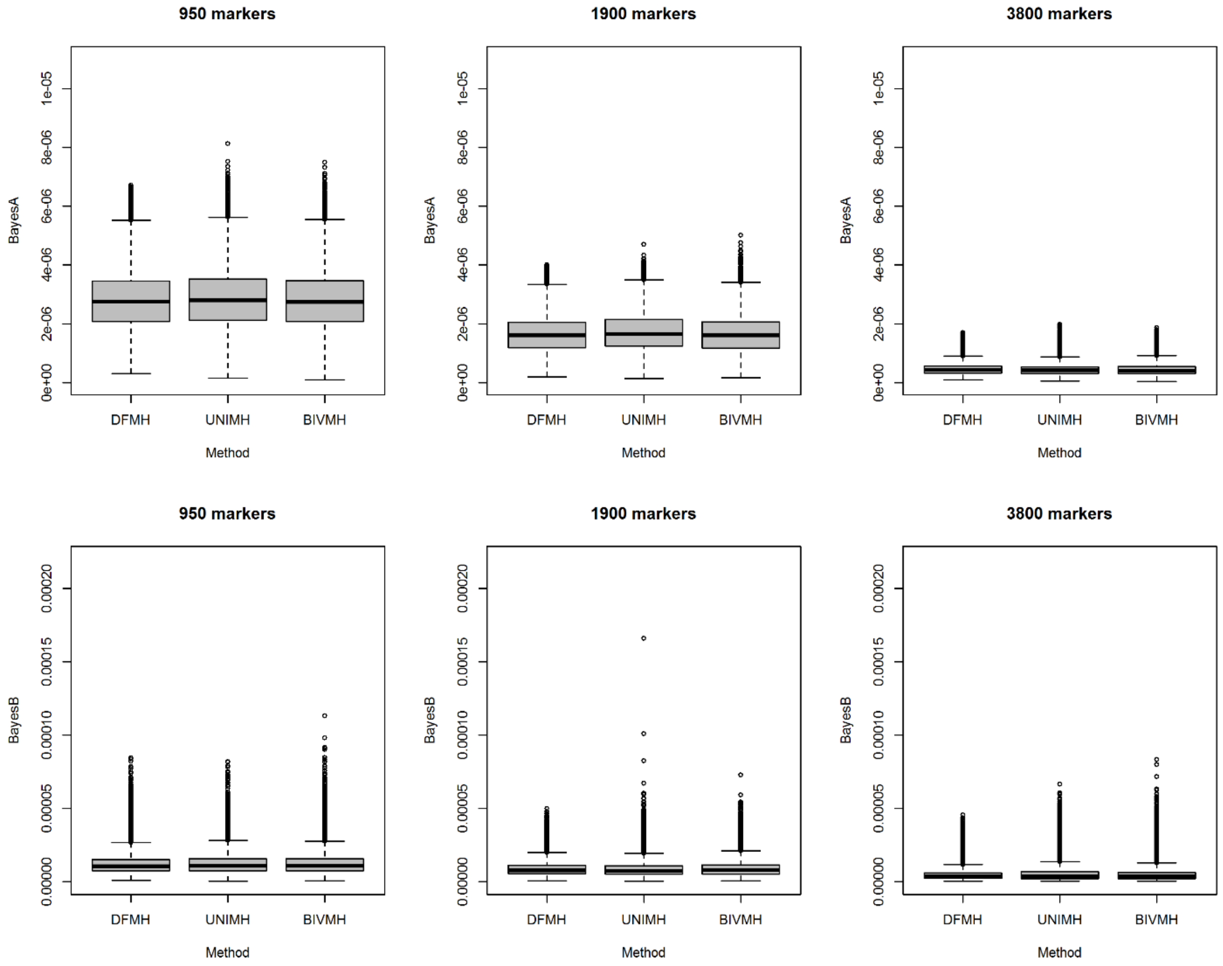

Figure S6: Boxplots of posterior densities of s^2 based on BayesA (top row) and BayesB (bottom row) analyses of mice body weights based on using 950, 1900 and 3800 markers under three different algorithms (DFMH, UNIMH and BIVMH).

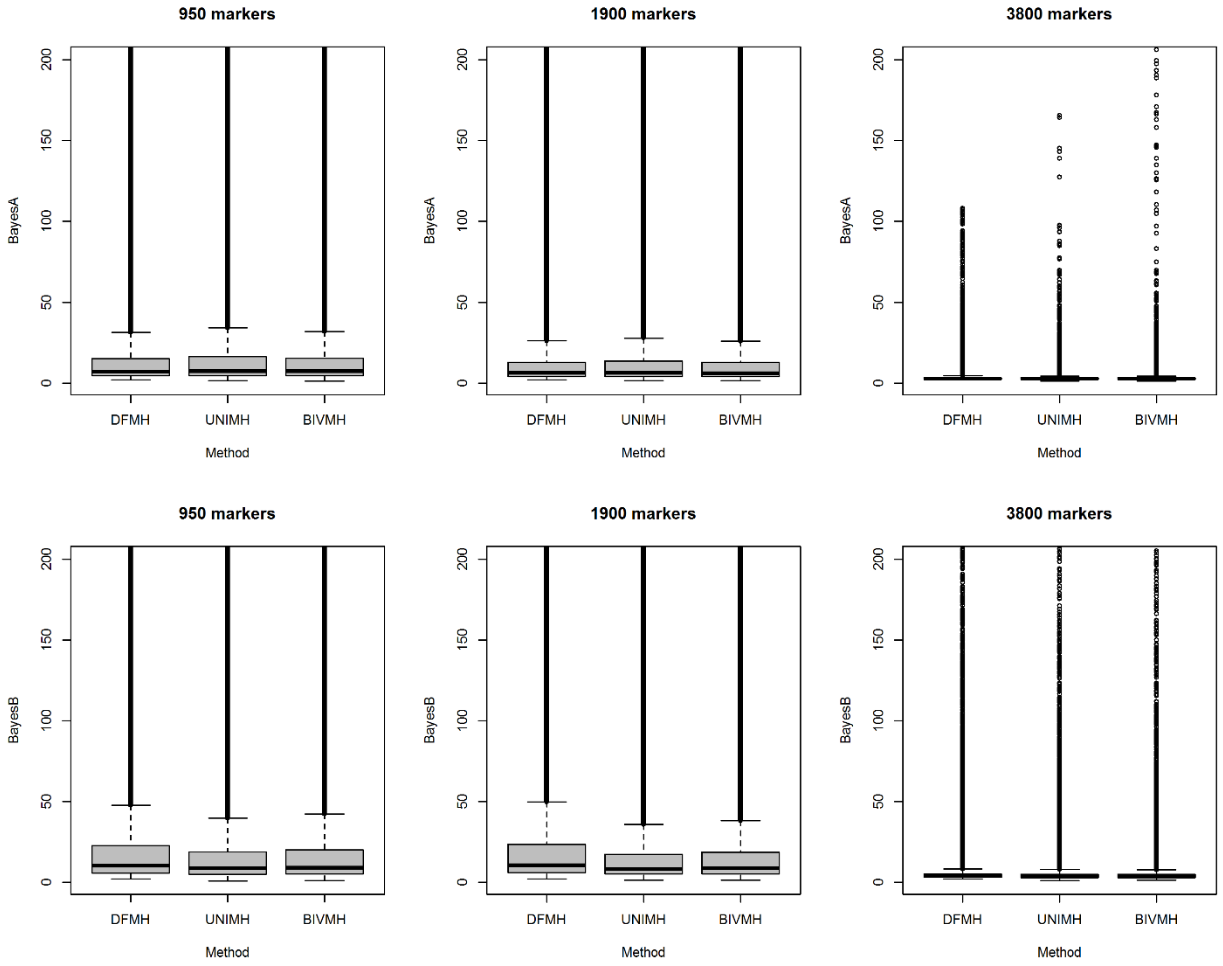

Figure S7: Boxplots of posterior densities of ν (ν <200) based on BayesA (top row) and BayesB (bottom row) analyses of mice body weights based on using 950, 1900 and 3800 markers under three different algorithms (DFMH, UNIMH and BIVMH).

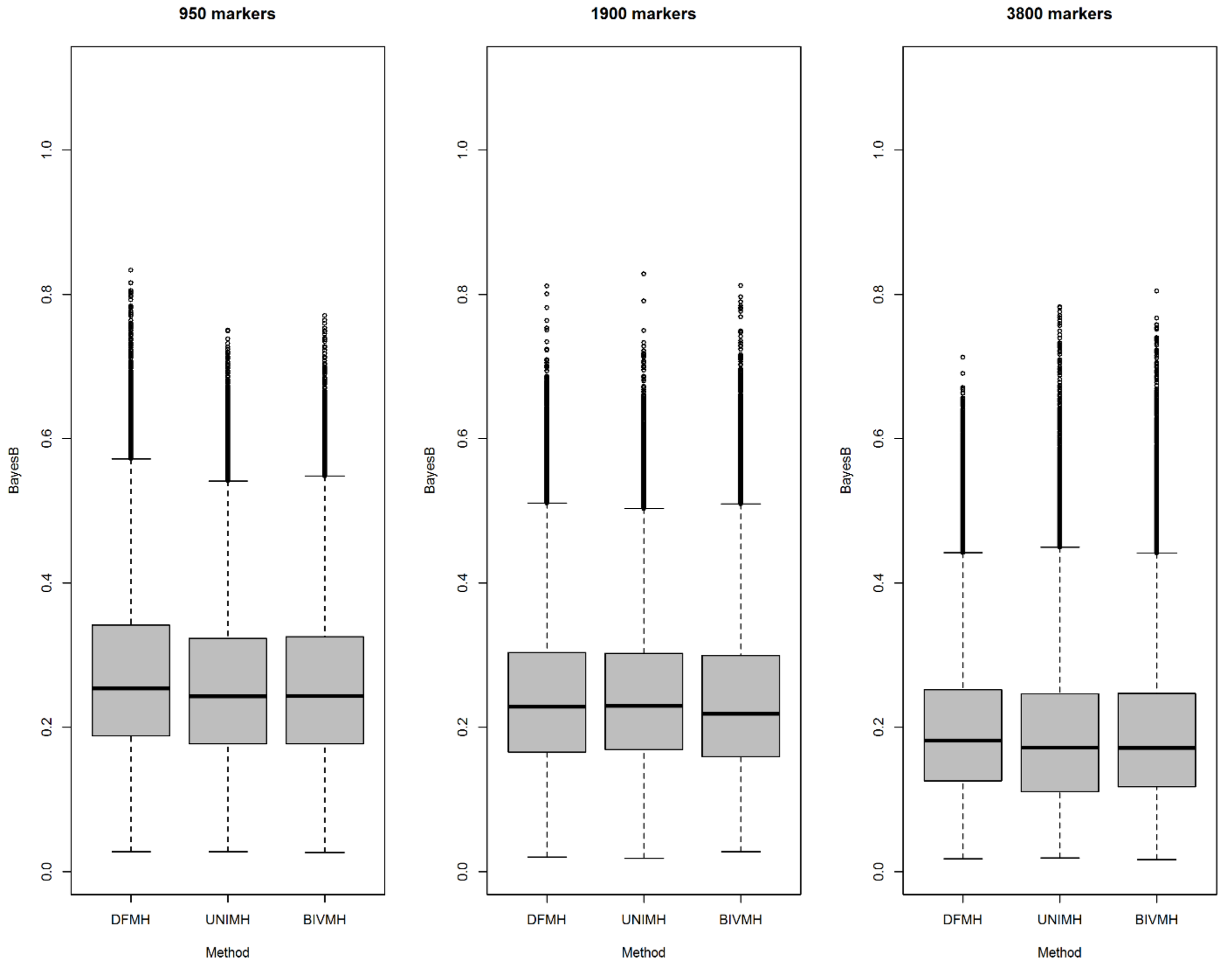

Figure S8: Boxplots of posterior densities of π based on BayesB analyses of mice body weights based on using 950, 1900 and 3800 markers under three different algorithms (DFMH, UNIMH and BIVMH).

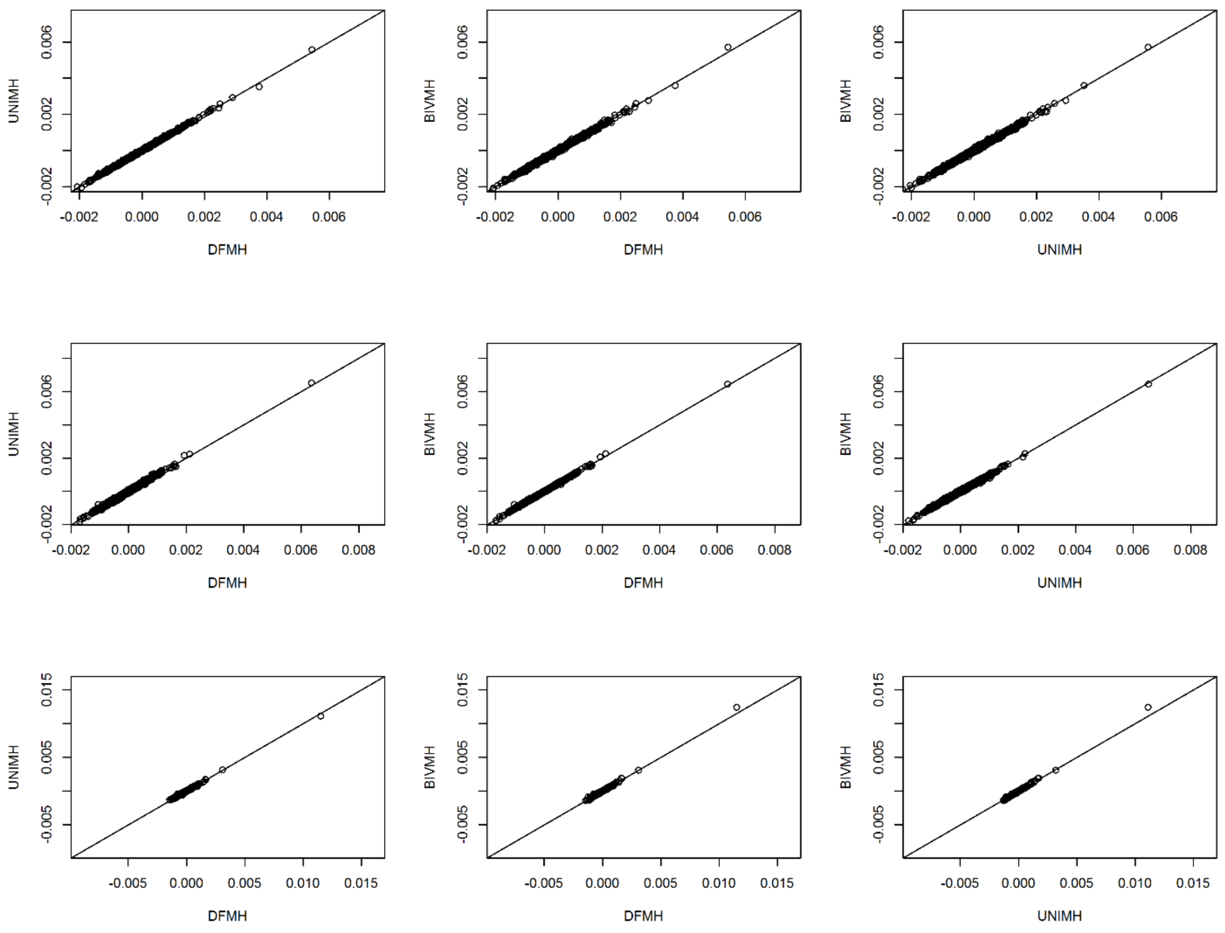

Figure S9: Scatterplot comparisons of posterior means of SNP effects under BayesA analyses of mice body weights using three different algorithms (DFMH, UNIMH and BIVMH) based on 950 markers (top row), 1800 markers (middle row) and 3800 markers (bottom row). Reference line of intercept 0 and slope 1 superimposed.

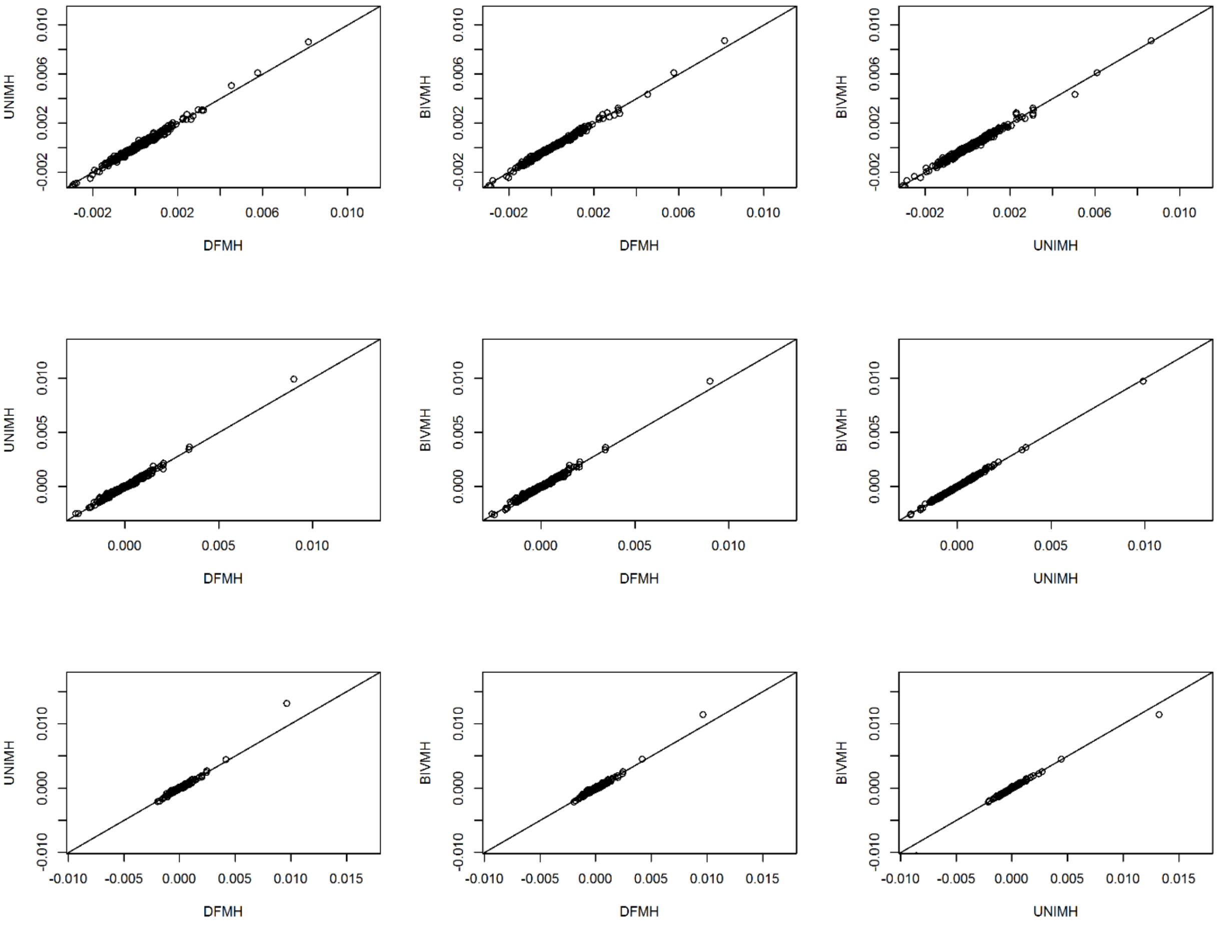

Figure S10: Scatterplot comparisons of posterior means of SNP effects under BayesB analyses of mice body weights using three different algorithms (DFMH, UNIMH and BIVMH) based on 950 markers (top row), 1800 markers (middle row) and 3800 markers (bottom row). Reference line of intercept 0 and slope 1 superimposed.

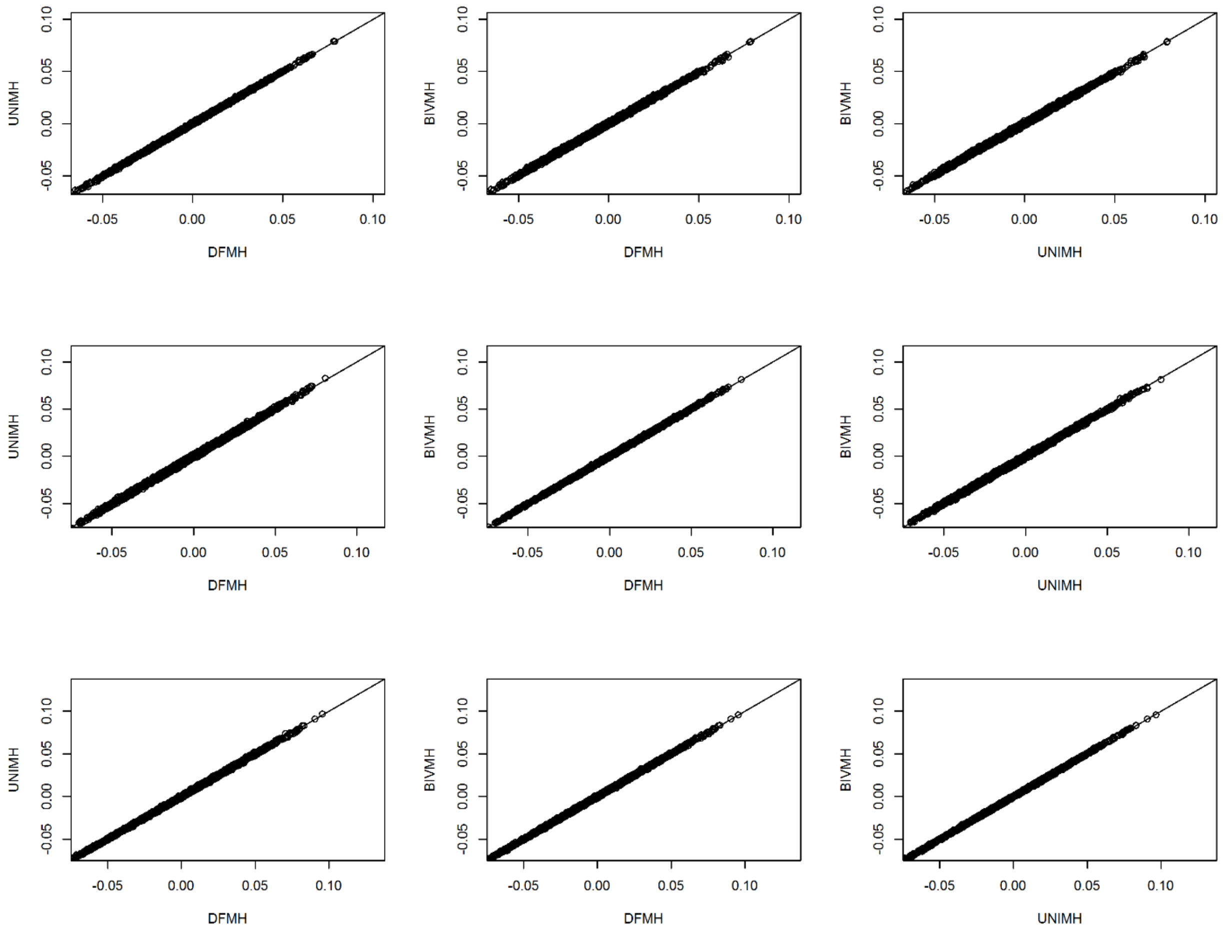

Figure S11: Scatterplot comparisons of posterior means of GEBV under BayesA analyses of mice body weights using three different algorithms (DFMH, UNIMH and BIVMH) based on 950 markers (top row), 1800 markers (middle row) and 3800 markers (bottom row). Reference line of intercept 0 and slope 1 superimposed.

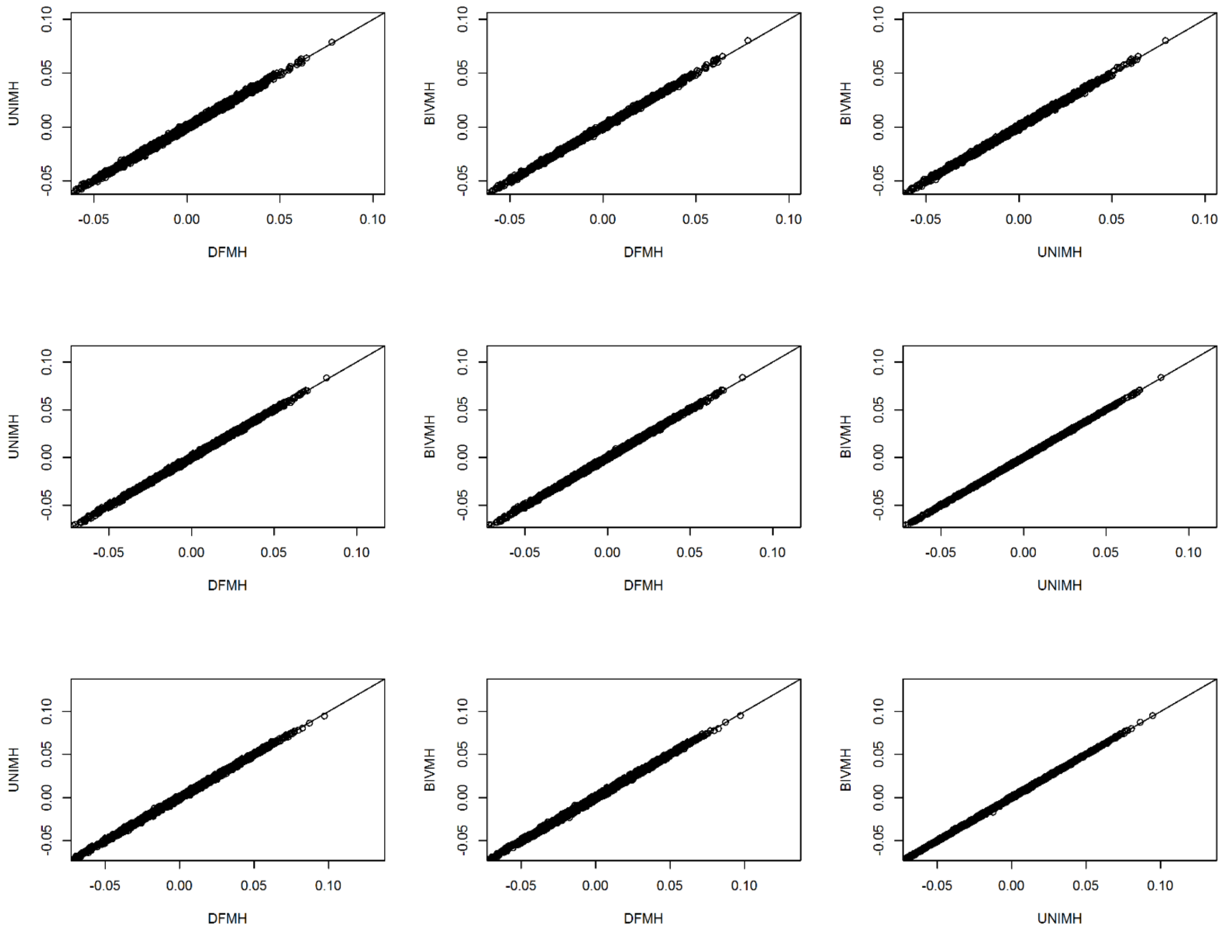

Figure S12: Scatterplot comparisons of posterior means of GEBV under BayesB analyses of mice body weights using three different algorithms (DFMH, UNIMH and BIVMH) based on 950 markers (top row), 1800 markers (middle row) and 3800 markers (bottom row). Reference line of intercept 0 and slope 1 superimposed.
